# Anorexia nervosa symptoms are induced after specific gut microbiota dysbiosis transfer in germ-free mice

**DOI:** 10.1080/19490976.2025.2563701

**Published:** 2025-11-15

**Authors:** Tristan Gabriel-Segard, Christine Heberden, Stanislas Mondot, Maeva Duquesnoy, Marika Dicembre, Laurent Naudon, Catherine Philippe, Elise Maximin, Anne Blais, Madalina Jacota, Nicolas Lapaque, Hervé M. Blottière, Stéphane Paul, Joël Doré, Sylvie Rabot, Mouna Hanachi

**Affiliations:** a INRAE, AgroParisTech, MICALIS Institute, Université Paris-Saclay, Jouy-en-Josas, France; b CIRI-Centre International de Recherche en Infectiologie, Team GIMAP, Université Claude Bernard Lyon 1, Saint-Etienne, France; c Clinical Nutrition Department, AP-HP Hôpital Paul-Brousse, University of Paris-Saclay, Villejuif, France; d UMR PNCA, AgroParisTech, INRA, Université Paris-Saclay, Paris, France; e Clinical Research Department, APHP University of Paris-Saclay, Le Kremlin Bicêtre, France

**Keywords:** Anorexia nervosa, germ-free mice, fecal microbiota transfer, undernutrition.

## Abstract

Anorexia nervosa (AN) is the most severe and life-threatening eating disorder. Its pathophysiology remains largely unknown, and no effective treatment currently exists for severe forms of the disease. Gut microbiota (GM) dysbiosis has been consistently reported in AN; however, no study has yet considered the role of the microbiota within the full spectrum of AN symptoms. To investigate the direct involvement of the microbiota in disease symptoms, we developed a murine model of fecal microbiota transplantation (FMT), using germ-free BALB/c mice colonized with fecal samples from well-characterized AN patients and healthy controls. Physiological, organ, and behavioral parameters were systematically monitored. We found that key AN-related features (including food restriction, anxiety-like behavior, physical hyperactivity, and elevated inflammatory responses) were transmitted to germ-free mice following transplantation with AN-derived microbiota. Likewise, organ-specific alterations associated with AN, such as liver dysfunction and disruption of ovarian follicles, were also reproduced. In conclusion, we demonstrate that the transfer of AN microbiota induces behavioral, physiological, and organ-level alterations reminiscent of the human disease. These findings highlight a major role of the gut microbiota in the symptomatology and progression of AN and open new therapeutic perspectives targeting this ecosystem.

## Introduction

Anorexia nervosa (AN) is the most serious eating disorder and affects 1.7%–3.6% of women,^1^ with a mean age of onset of two peaks: 15–16 y and 22–24 y.^2^^,^^3^ The disease burden is notably reflected by a high mortality rate, up to 16 times greater in patients with the most severe and chronic forms than in the general population.^4^ Somatic complications and suicide are the two first causes of death.^5^ AN is known to chronically evolve in 23% of cases^6^ in the remitting and relapse phases and is associated with severe undernutrition, requiring specialized nutritional care with transitory enteral nutrition.^7^ According to the fifth edition of the Diagnostic and Statistical Manual of Mental Disorders,^8^ AN is defined as an inability to maintain adequate dietary intake to maintain a normal weight for age, associated with an intense fear of gaining weight or becoming fat, or persistent behavior that interferes with weight gain and disturbances in body image. Two types of AN are described: the pure restricting type (AN-R) and the binge-eating/purging type (AN-BP), defined by cycles of large meals followed by purging behaviors (vomiting and/or laxative abuse).^8^ Anxiety, depressive symptoms, and problematic use of physical activity are often associated with this symptomatology.^9^ Somatic complications of AN are frequent and can be the consequence of malnutrition, purging behaviors, or adverse effects of refeeding. The pathophysiology of AN is complex and multifactorial: i) genetic theory,^10^ which involves family vulnerability and heritability; ii) the endocrine hypothesis, which involves disturbances of hormones (neuropeptide Y, leptin, and ghrelin) that regulate hunger and satiety;^11^ iii) certain hypotheses, which suggest abnormalities located in the reward pathways of the brain, bringing AN closer to addictive disorders;^12^ and iv) psychodynamic functioning issues in the fields of attachment, separation, and refusal of individuation, exit from childhood, or loss of child omnipotence.^13^ The dysbiosis between the host and its gut microbiota (GM) is a promising etiopathological pathway.^14^^,^^15^ Compared with healthy controls, studies conducted on GM samples from patients with AN have reported significant differences over the last 10 y.^16^ GM *α*- and β-diversity are not homogeneous across studies. Comparing patients with AN to a normal-weight group of subjects revealed specific characteristics: an increased abundance of bacteria from the Bacillota phylum and a decreased abundance from the Bacteroidota phylum.^14^^,^^16^^,^^17^ A decrease in butyrate-producing species has been reported at the genus level, including a significant decrease in the *Roseburia* genus.^16-19^ Nutrition during inpatient care impacts the microbiota by modulating the abundance of some bacterial families, marked by a relatively low abundance of Coriobacteriaceae and a relatively high abundance of Ruminococcaceae.^16^^,^^17^ Interestingly, refeeding does not normalize GM diversity or abundance in patients compared with controls.^20^ Anxiety and depression are comorbid symptoms associated with GM dysbiosis^21^ especially in AN patients. One study revealed a negative correlation between the Beck Depression Inventory score and the abundance of *Clostridium* spp.^22^ To the physiological features of AN, *Methanobrevibacter smithii* abundance is reported to be negatively correlated with body mass index and is overrepresented in patients with AN, such as *Anaerostipes*, *Anaerotruncus,* and the *Akkermansia* genus of mucin-degrading bacteria.^16^^,^^17^ Hepatic cytolysis (marked by hypertransaminasemia) and enterocyte suffering (marked by low blood citrulline concentration) are significantly associated with a high abundance of Flavobacteriaceae in severely undernourished patients with AN.^7^ Garcia et al. reported that 10 of 14 studies presented inconsistent results concerning *α*-diversity and *β*-diversity in subjects suffering from AN.^16^ Comparisons between studies remain difficult, as the patients recruited are not accurately phenotyped concerning the stage and characteristics of AN. AN groups appear unequal in terms of age, method of diagnosis, type of AN, body mass index (BMI), and comorbidities or complications, and this information is poorly documented. Recently, Fan et al. conducted a comprehensive analysis associating metagenomic and metabolomic data with disease traits evaluated using the Eating Disorder Inventory-3 (EDI-3). They inferred a causal linkage between 13 microbial features as initiators, 11 blood metabolites as mediators, and 7 EDI-3 dimensions as outcomes.^19^ The immune system, at the interface of the host and the intestinal ecosystem, was characterized in human subjects with AN. Results are not consistent, revealing, in a meta-analysis, an elevation of pro-inflammatory cytokines such as interleukin-1β (IL-1β), interleukin-6 (IL-6), and tumor necrosis factor-alpha (TNF-*α*) in subjects with AN,^23^ as found in patients with ED.^24^ Whereas most recent longitudinal studies do not confirm the previous results regarding those cytokines, revealing no differences compared to healthy controls.^25-27^ These studies suffer from poor precision of the AN phenotype of participants. To address this issue, our group designed a study about long-lasting anorexia nervosa in women. It analyzed cytokines and cellular phenotype in blood, identifying a non-inflammatory profile associated with regulatory T cells in patients presenting with a chronic form of the disease.^28^ To our knowledge, no studies have highlighted the associations among specific immune characteristics, abnormal enterohormone concentrations, and microbiota analysis. The germ-free mouse model is useful for identifying the physiological consequences of transferring a specific microbiological ecosystem.^29^ To date, three studies have been conducted using a gnotobiotic mouse model based on the transfer of fecal microbiota from patients suffering from AN.^19^^,^^30^^,^^31^ Taken together, the results revealed characteristic symptoms of anorexia nervosa: increased anxiety behavior,^30^ impaired weight gain^19^^,^^30^ and reduced food efficiency.^19^^,^^30^ The GM appears to be a significant factor in mediating the emergence of AN features.

To achieve these results, we hypothesized that the transfer of fecal microbiota would widely impact different aspects of host physiology and the transmission AN symptoms to germ-free mice, such as food consumption, weight gain, body composition, behavior (physical activity, anxiety-like, and depression-like manifestations), enteroendocrine functions, mucosal immunity, and ovarian and liver physiology. As the individual characteristics of donor patients are poorly documented in studies of fecal microbiota transfer, we provided a high level of precision to generalize our results with greater relevance. Recruited patients have a long history of AN, and stool sampling is carried out during the acute phase of undernourishment. In line with our commitment to high precision, patients underwent complete phenotyping of disease characteristics and biological parameters, representing the first study of fecal microbiota transfer in ANs with this level of precision.

## Materials and methods

*Clinical characteristics of gut microbiota donors.* Three women admitted for inpatient nutritional treatment at the Clinical Nutrition Department of Paul Brousse University Hospital (APHP, Villejuif, France) were recruited in January 2021. Patients were, on average, 29 y old (32, 27, 29 y old). All patients received a diagnosis of pure restricting-type anorexia nervosa after submission to the Eating Disorder Diagnosis Survey French-validated version^32^ criteria, and a clinical structured interview from the department's psychiatrist. The self-reported disease onset history resulted in a mean duration of disease of 14.7 y (19, 12, and 13 y). Patients were recruited according to the following inclusion criteria: female aged 18 y or older, with a body mass index (BMI) of 15 kg/m² and hospitalized as nutrition inpatients. The exclusion criteria were i) antibiotics taken in the two months preceding hospitalization,^33^ ii) purging behavior with laxative abuse in the month before hospitalization,^33^ iii) somatic comorbidities that may be associated with a change in the GM profile (diabetes, irritable bowel syndrome, or other digestive pathology, metabolic disease), and iv) a history of overweight or obesity.

All the subjects provided informed written consent for inclusion before participating in the study. Healthy volunteers were recruited based on their age (over 18 y), BMI (between 18.5 and 25 kg/m²), absence of metabolic abnormalities, and a recent weight change. The study was conducted in accordance with the Declaration of Helsinki and was part of the INT-METAVOSA protocol approved by the Ethics Committee of CPP (Comité de Protection des Personnes) APHP211375. Patients and their disease conditions were evaluated according to current practices in the department and international recommendations^34^^,^^35^ to achieve a precise phenotyping and constitute the research group.

The following parameters for each patient and control were assessed: weight, height, calculated body mass index (BMI: weight/height^2^), biological nutritional parameters (albuminemia, transthyretin, and C-reactive protein), and nutrient intake.

Psychometric symptom scales were applied to the subjects to assess AN symptomatology and comorbidities. Intensity of eating disorder is measured by the Eating Disorder Inventory (EDI-II) total score and subscales. The Beck Depression Inventory (BDI) and the Hospital Anxiety and Depression Scale (HAD-S) are used to estimate the intensity of symptoms from anxiety and depression disorders. Associated symptomatology to AN in the field of obsession and compulsion is measured by the Maudsley Obsessional Compulsive Inventory (MOCI),^36^ and for social phobia symptomatology using the Liebowitz scale. The intensity of physical activity is estimated using the Godin Exercise Scale. Intestinal symptomatology typically encountered in AN is evaluated using the Francis score for irritable bowel syndrome symptomatology, and stool consistency is assessed to inform the diagnosis of constipation or diarrhea symptoms. (Additional material: Supplementary Table 1).

*Microbiota fecal transfer preparation and FMT.* Ten grams of feces from whole stool sample collected from Stool collector devices were provided by MaatPharma® (Lyon, France) were homogenized using a cryoprotectant media and transferred into Stomacher Filter Bags (Seward BA6041/8TR or VWR 432−3119, with 0.5 mm holes) within an anaerobic chamber. Four milliliters of maltodextrin-trehalose mixture was added per gram of stool.^37^ Five-minute hand mixing through the filter bags (closed by clips, VWR 432−3116) ensured homogenization and filtration. The suspension was subsequently aliquoted and stored at −80 °C. The day of gavage, thawing was performed for 5 min in a water bath at 37 °C. Aliquots from the three AN donors or the three control donors were pooled before administration to the mice by oral gavage within an anaerobic chamber.^38^

Ethics Committee of the INRAE Research Center at Jouy-en-Josas and authorized by the Ministry of Research (authorization reference: APAFIS#22735−201910161203326 v3). BALB/c mice were provided by the breeding unit of the Anaxem germ-free animal facility (INRAE, Micalis Institute, Jouy-en-Josas, France). The mice were fed a chow diet with *γ*-irradiated 45-kGy pellets (R03; Scientific Animal Food and Engineering, Augy, France) and autoclaved tap water*.* Two groups of 12 female mice were housed in two flexible film isolators for the first 3 weeks of microbiota implantation and then transferred to individual cages until the end of the experiment. Transparent plastic tunnels, chewing sticks, and nesting material were placed in each cage for environmental enrichment. The mice were exposed to an artificial light of 100 lux (with a 12-h light cycle, light phase 7 am–7 pm) and a temperature between 20 and 24 °C. Throughout the entire protocol, four male mice were housed in a cage within the isolator to create a pheromone environment and synchronize the estrus cycle of the females. A test to ensure the germ-free status of the mice was performed 3 d before FMT using microscopy, aerobic culture, and anaerobic culture in Sabouraud, LCY and LB media.

Four-week-old germ-free mice received 0.2 ml of the pooled human fecal suspension by oral gavage immediately after they arrived in the experimental isolators, followed by a second aliquot of the identical mixture 2 d later. The mice were then housed for 3 weeks without any experimental intervention, with free access to food and water (ad libitum). The period is required to allow the microbiota to be implanted and stabilized. One mouse per group died during this time, one in the control group after the gavage, and the other in the AN group due to a water dispenser issue. Individual fecal samples were collected for 16S rRNA gene sequencing analysis 3 weeks after FMT (T1), 4 weeks later (T2), and before euthanasia at week 8 (T3); these samples were also used to analyze intestinal biological parameters (ZO-1, calprotectin, IgA, and IgM). The stool was collected, weighed, and immediately frozen. The mice's weight and food consumption were monitored twice a week until the end of the protocol. During weeks 6 to 8, behavioral tests were conducted in the isolator to assess anxiety-like (open-field test, novelty object test, and step-down test) and depression-like (splash test) behaviors. The number of estrous cycles was analyzed from week 4 to 9 (36 d), to assess the effect of microbiota on sexual physiology (>4 estrous cycles per mouse). The estrous cycle of the mice was evaluated via vaginal washing and cytologic analysis of epithelial cells. At the end of the eighth week of the protocol, two other behavioral tests for assessing depression-like behavior (forced swim) and anxiety-like behavior (elevated plus maze) were carried out outside the isolators, as the devices were too large to be accommodated in the isolators. The mice were euthanized by decapitation immediately after these tests. The truncal blood was collected in EDTA (0.5 M)-precoated tubes for FACS assays and in tubes containing separating gel. The gel tubes were centrifuged (3000 × *g*, 20 min, 4 °C) to obtain serum samples, which were then aliquoted into cryotubes and stored at −80 °C. Intestine pieces were collected, gently washed with cold PBS, and stored in PFA 4% and cell recovery solution (Corning). Body composition (bone, fat, and lean mass) was measured in mouse carcasses via dual X-ray absorptiometry using a Lunar PIXImus densitometer (DEXA-GE PIXImus; GE Lunar Corp., Madison, WI, USA) after dissection and extraction of tissue for sampling. The measurement of a phantom controlled the stability of the device before each session. The images were analyzed using the software provided by the device (Lunar PIXImus v2.10; GE Lunar Corp.) via auto-thresholding. The PIXImus was calibrated using an aluminum/lucite phantom (corresponding to a bone mineral density of 0.0592 g/cm² and 12.5% fat) on each testing day, according to the manufacturer's instructions.

*16S rRNA gene sequencing.* Bacterial DNA was extracted from the stool samples using the NucleoSpin DNA Stool Kit (Macherey-Nagel–740472) as recommended by the manufacturer. The 16S rRNA gene was amplified via PCR with primers targeting the V3V4 region (341F: CCTACGGGNGGCWGCAG, 805 R: GACTACHVGGGTATCTAATCC) via the IMR (https://imr.bio/index.html). The PCR conditions and sequencing library preparation are described in detail at https://www.protocols.io/workspaces/integrated-microbiome-resource-imr/publications. The amplicon library was sequenced on an Illumina MiSeq platform via a 2 * 300 bp V3 kit. Any remaining adapter and primer sequences were trimmed, and the reads were checked for quality (≥30) and length (≥250 bp) using Cutadapt.^39^ The reads were further corrected for known sequencing errors via SPAdes^40^ and then merged via PEAR.^41^ The Vsearch pipeline ^42^ was applied to dereplicate (–derep_ prefix–minuquesize 2) and cluster (–unoise3) the merged reads, as well as to check for chimeras (uchime3_denovo). Taxonomic classification of ASVs was performed via the classifier from the RDPTools suite.^43^ The sequencing data are deposited in the NCBI BioProject platform under the accession number PRJNA1075908. Microbiota sequencing was performed on fecal samples 3 weeks after the first gavage (T0), corresponding to the phase of microbial implantation, 4 weeks after microbial implantation (T1), and at the end of the protocol (T2).

*Scoring of behavior in transplanted mice.* All tests were performed between 10 am and 1 pm to avoid hours after the light–dark transition corresponding to the first 6 h of the light cycle.^44^ Each test was conducted on all mice within 2 d, in a random order determined by the number of passages. The first set of four tests was performed within the isolator over a period of 3 weeks, in the following order: open field (OF), step-down (SD), splash (SP), and novelty object (NwO) tests. A set of tests was carried out successively in a conventional dedicated room before each mouse was euthanized: the elevated plus maze (EPM) test and the forced swim test (FST). The mice were removed from the isolators and housed in a conventional room for 2 h to allow them to adapt before the test.^45^ All tests were recorded, and experienced observers manually analyzed the videos using ANY-maze 7.2 software (Stoelting Co., Dublin, Ireland), blind to the category. We then applied z-normalization across data obtained in the six behavioral tests, as described previously by Guilloux et al.^46^ The Z-scores indicate how many standard deviations (*σ*) an observation (x) is above or below the mean of a reference group (µ), i.e., Z-score = (x–µ)/σ.^47^ Mice from the gHC group that received healthy donor fecal microbiota were chosen as the reference group. Z-scores for the physical activity index were computed across the tests (distance traveled, number of rearing, and speed for both OF and NO) for equal weighting. Anxiety was described using dimensions to consider in the EPM test (anxiety index calculated as Cohen^48^) and in the NO global Z-score (combining the Z-score of mean visit time and the Z-score of latency to first visit). The OF anxiety dimension was not considered as the OF arena is not regularly equipped with high walls and lighting in the center.

*Open field: The mouse was gently placed using a tube in the center of an open cage (43 cm long, 27 cm wide, 22 cm high) for 15 min and videotaped. The traveled distance, the number of rearing, and the number of the center crossing were recorded and analyzed. We chose this test to assay physical activity by measuring the total length traveled, the number of rearings, and the speed, which is interpreted as a better ability to explore the environment.^49^ The material was cleaned with a water-wet cloth after each mouse was tested. This test was performed within the isolator. Z scores were calculated and computed with Z scores of the same dimension from the novel object test to calculate the physical activity index.

The constraints of the isolator do not allow the use of an optimal OF arena in our protocol. In feet, the walls and lighting do not conform to standard practice, and do not provide the intense light and shade required for anxiety measurement. In order to maintain caution in interpreting the results. We only considered the distance covered by the animals and not the behavioral elements associated with anxiety, i.e. the number of center crossing and the time spent in dark and lighted areas.

*Novelty object test: The mouse was placed at a distance of an unknown object in a dimly light cage (43 cm long, 27 cm wide, 22 cm high), whose floor was divided into 4 × 7 squares. The novelty object test is commonly used to assess anxiety, as fewer visits and short visit duration are interpreted as anxious behavior. An unknown object build with white construction bricks was placed 7.5 cm from one of the short sides of the rectangular arena. Each mouse was placed facing the opposite wall. The time to go exploring the object (latency time), the time spent exploring the object (i.e., the nose directed towards the object at a distance of less than 2 cm), and the locomotor activity (distance traveled, speed, rearing number) were monitored for 10 min.^50^ The material was cleaned with a water-wet cloth after testing each mouse. This test was performed within the isolator. Mean visit time is calculated by dividing the time spent exploring by the number of visits. We combine the results of the Z score mean visit time and Z score latency time to explore the NwO and calculate the global Z score.

*The step-down Test: This test was chosen to measure the anxiety dimension of mice's behavior and is adapted from previous article.^51^ It was designed to assess the readiness of the mouse to escape from an elevated place by stepping down onto a horizontal surface, here a 4 cm high platform (15 cm long and 10 cm wide) made of Lego®. Each mouse was gently put on the platform, and the latency to step down with all four paws was recorded during three trials of 5 min, each with a 1 min interval. After testing each mouse, the platform was cleaned with a water-wet cloth. This test was performed within the isolator. Results are expressed in the Z score and interpreted as more anxiety in case of no descent from the platform or a higher latency for stepping down with the four paws within a 300-s timelapse.

*Splash test: This test assessed the apathy dimension corresponding to depression-like behaviors. A 10% sucrose solution was sprayed on the dorsal coat of the mouse in its home cage. The latency before the first grooming attempt and the overall time of grooming behavior were measured for 5 min.^52^ This test was performed within the isolator.

*Elevated plus maze: This test measures anxiety in rodents, exploiting their natural aversion to backlit areas. The test apparatus consists of a gray maze in the shape of a cross composed of four arms (30 cm long, 5 cm wide). Two opposite arms were surrounded on three faces by 16 cm high walls (closed arms), while the other two were open (open arms). The maze was raised 50 cm from the floor and illuminated by room light. At the beginning of the test, the mouse was gently placed using a tube in the intersection square, facing an open arm, which was considered an aversive area. The number of visits (entries and failed entries) and the time spent in the open and closed arms were recorded for 6 min. The results were expressed as the ratio of the number of visits (or the time spent) in the open arms over the overall number of activities (or time spent) in both closed arms and open arms. An “entry” was considered as such if the mouse put all four paws in the open or closed arms. If the mouse put only the two front paws, this was considered a “failed entry”.^53^ The anxiety index is calculated from the EPM test dimensions according to the Cohen et al. 2013 formula. This test was performed without the isolator in a dedicated room following an environmental habituation of 2 h. The material was cleaned with a wet cloth after testing each mouse.

*Forced swim test. This test evaluates the resignation dimension of behavior in a highly stressful condition. Time of immobility after gently putting off in the water is interpreted as a despair manifestation related to a depressive condition.^54^ This test is considered a severe animal procedure and is closely monitored to ensure compliance with ethical principles. The mouse was gently placed in a glass cylinder (30 cm high) filled with 20 ml of 23−25 °C water. The mouse was forced to swim for 5 min, and the test was videotaped. The immobility time of each animal was monitored. The mouse was judged to be immobile when it remained floating motionless and kept its head above the water's surface. The water was changed between each test.

*Monitoring biological parameters after FMT.* The concentrations of cytokines and enterohormones in the serum were measured via Luminex® multiplex technology. A commercial kit Pro Mouse Cytokine 23-Plex (IL-1α, IL-1β, IL−2, IL-3, IL-4, IL-5, IL-6, IL-9, IL-10, IL-12 (p40), IL-12 (p70), IL-13, IL-17A, Eotaxin, G-CSF, GM-CSF, IFN-*γ*, KC, MCP-1 (MCAF), MIP-1α, MIP-1β, RANTES, TNF-α; M60009RDPD, Bio-Rad) was used for cytokine assays in serum, which were performed in duplicate with no serum dilution—custom commercial kits for ghrelin, leptin, PYY, and GLP1 (MMHMAG-44K-04, Merck). Serum corticosterone was measured using a commercial ELISA kit (MBS2505570, MyBioSource, San Diego, USA) without sample dilution. A workable solution was made to assay fecal biological parameters by solubilizing the feces pellet to 100 mg/ml in PBS with a Halt^TM^ protease inhibitor cocktail (78,438, Thermo Fisher Scientific). The supernatant was then recovered after centrifugation and used for further analysis. Zonulin assays were performed in feces diluted 1:2000 using a commercial kit (MBS2603528, MyBioSource, San Diego, USA). Fecal calprotectin was quantified via ELISA using the DuoSet Mouse S100A8/S100A9 Heterodimer Kit (DY8596-05, R&D Systems, Minneapolis, USA) according to the manufacturer's recommendations. At the time of the assay, the stool was solubilized in extraction buffer (0.1 M Tris, 0.15 M NaCl, 1.0 M urea, 10 mM CaCl2, 0.1 M citric acid monohydrate, and 5 g/L BSA (pH 8.0)) and then centrifuged for 5 min at 3000 × *g*. Immunoglobulins A and M were measured in serum (diluted to 1:4000 and 1:100, respectively) and in fecal solution (diluted to 1:2000 and not diluted, respectively) via commercial kits (MBS564073 for IgA and MBS2514903 for IgM; MyBioSource, San Diego, USA). All ELISA tests were performed in duplicate, and the average of the results was used for analysis. SCFAs were analyzed on a gas–liquid chromatograph (Autosystem XL; Perkin Elmer, Saint-Quentin-en-Yvelines, France) after water and protein extraction with phosphotungstic acid”.^55^

For the intestinal cell suspension, three intestinal pieces, each 1 cm in length, were cut, soaked in cell recovery solution (Corning), and stored on ice overnight at 4 °C. The tubes were then shaken vigorously for 1 min. The tube content was filtered and scraped through a 200 µm strainer. The cell mixture was then filtered through a 70 µm strainer. The contents of the filter were pipetted and stored on ice. The quality of digestion is ensured by checking the presence of villi under an optical microscope. The mixture was subsequently centrifuged (140 × *g*, 10 min, 20 °C). The cell pellet was treated with trypsin for 5 min at 37 °C. Cells were then washed and maintained in RPMI medium (Roswell Park Memorial Institute) supplemented with 10% fetal bovine serum until the time of analysis.

*Immunophenotyping after FMT.* Lymphocyte populations were analyzed by flow cytometry. All protocols were performed with a BD FACSCanto II (BD Biosciences). Compensations were made via the VersaComp Antibody Capture Kit (Beckman Coulter Inc.). The compensation matrix and data analysis were performed via Kaluza Analysis Software 1.3 (Beckman Coulter Inc.). Three panels were constructed to reach all the populations of interest (Supplementary Mat & Met File). Panels 1 and 2 were performed in blood samples and intestinal cell suspension, and Panel 3 was completed only in intestinal cell suspension. Panel 1 used the commercial BD Cytofix/Cytoperm Plus Fixation/Permeabilization Solution Kit with BD GolgiPlug (BD Biosciences) and leukocyte activation cocktail (BD Biosciences) at a concentration of 2 µL/mL of cell mixture at 10^6^ cells/mL. Following the kit's recommendations, the cells were stimulated for 4 h at 37 °C. Panel 2 used the BD Pharmingen Mouse FoxP3 Buffer Set (BD Biosciences) commercial kit. Cell staining included CD3e BV510 (563024, clone 145-2C11, BD Biosciences), CD4 APC-H7 (560181, clone GK1.5, BD Biosciences), LPAM−1 A647 (562376, clone DATK32, BD Biosciences), CD45 APC (559864, clone 30-F11, BD Biosciences), CD69 BV421 (562920, clone H1.2F3, BD Biosciences), CD127 BV421 (562959, clone SB/199, BD Biosciences), IFN-*γ* PE-Cy7 (557649, clone XMG1.2, BD Biosciences), TCRγ/δ PE-Cy7 (118123, clone GL3, BioLegend), CD11b PE-Cy7 (552850, clone M1/70, BD BioSciences), IL-17a A488 (560220, TC11-18H10, BD BioSciences), CD8a FITC (553030, clone 53−6.7, BD BioSciences), Vβ8.1−8.2 TCR FITC (553185, clone MR5−2, BD BioSciences), IL−9 PerCP-Cy5.5 (561492, clone D9302C12, BD Biosciences) and IL−4 PE (562044, clone 11B11, BD BioSciences). Concentration for staining were used according to manufacture recommendations for 20 min at RT in dark.

*Histological analysis.* Vaginal smears were obtained daily between 8:00 and 9:30 am by rinsing the vaginal cavity with 50 µL of sterile PBS using a pipette tip. The suspension was collected and smeared on a glass slide. When the smear was dry, the cells were colored with a solution of violet cresyl (Sigma). The different proportions of nucleated epithelial cells, cornified cells, and leukocytes determined the cycle's multiple stages, as described by Cora et al.^56^

The ovaries and liver were collected, weighed, and stored for histological analysis. The ovaries were removed, fixed in 4% paraformaldehyde for 24 h, and transferred to 70% ethanol. For hematoxylin-eosin (H&E) staining, the ovaries were embedded in paraffin and sectioned at a thickness of 5 µm. After deparaffinization and rehydration, the sections were stained with H&E. The follicles in each ovary were counted every tenth section. One slide was mounted with 10 to 15 sections, and four slides were counted for each ovary. Follicles with visible nuclei were scored and classified according to stage.^57^ A section of the liver, taken from the same hepatic lobe of each mouse, was fixed in 4% paraformaldehyde 24 h and transferred to 70% ethanol. The samples were embedded in paraffin and sectioned at 5 µm and then subjected to H&E staining. The sections were also stained with Black Soudan B to detect lipofuscin and counterstained with methyl green. All the histological sections were scanned using the Panoramic SCAN system (@BRIDGe histological facility, INRAE, Jouy-en-Josas) and analyzed using CaseViewer software to determine cell sizes.

*Statistical analysis.* Microbiota composition analysis was performed via R programming language and software (R Development Core Team 2012), specifically via the packages RColorBrewer (v 1.1-3), plots (v3.1.3), gdata (v2.19.0), xlsx (v0.6.5), ade4 (v1.7-22), vegan (v2.6-4), reshape2 (v1.4.4), and PMCMRplus (v1.9.6). The ASV counts were normalized via simple division according to their sample size and then multiplied by the size of the smallest sample. *α* diversity and richness were estimated via the ASV table data and the functions “diversity” and “estimateR”. A distance matrix for *β* diversity analysis was computed via the “vegdist” function and the Bray‒Curtis method. Principal coordinate analysis was conducted using “dudi.pco” on the distance matrix data. Kruskal‒Wallis rank sum tests and post hoc pairwise Wilcoxon rank sum tests were used to detect differences between groups of variables. *P* values were corrected as necessary via false discovery rate correction.

For other datasets, normality was tested via the D’Agostino and Pearson normality tests. Normally distributed data with equal group variances is expressed as the mean and standard deviation (SD). Non-normally distributed datasets, or datasets with unequal group variances, are described as medians (interquartile ranges). The level of significance was set at *p *< 0.05 (*p < 0.05, **p < 0.01, ***p < 0.001). Comparisons of means were conducted using the adapted method, which accounts for the normality of the dataset. Student's t-test was conducted in the case of a normal distribution, with the level of significance set at *p *< 0.05 (*p < 0.05, **p < 0.01, ***p < 0.001). The Mann‒Whitney test (compare ranks) was conducted in the case of a non-normal distribution detected by the D'Agostino and Pearson normality tests, with the level of significance set at *p *< 0.05 (*p < 0.05, **p < 0.01, ***p < 0.001). In the case of multiple comparison tests, q values are presented (Figure 5A), with a false discovery rate of 5% and a low maximum number of multiple tests (*n* = 6). We used two-stage step-up (Benjamini, Krieger, and Yekutieli) analysis methods.

**Figure 1.**
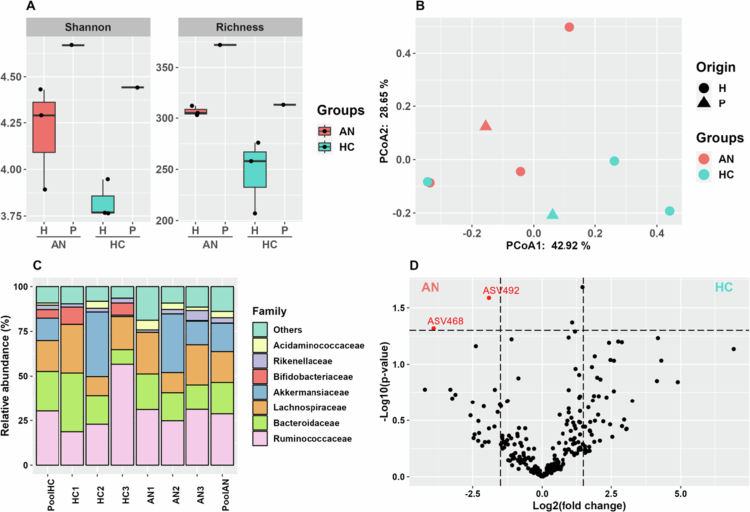
Analysis of the microbiota composition of human donors. A: Gut microbiota diversity and richness observed for AN patients and HC volunteers and their respective fecal pools. Wilcoxon test: Shannon–*p*-value = 0.19; Richness–*p*-value = 0.08. B: Principal coordinate analysis (PCoA) assessing the microbiota dissimilarity of human and pooled fecal samples. C: Bar plot displaying the relative abundance at the bacterial family level in each group and their respective pools. D: Volcano plot depicting ASVs whose abundance significantly differed between AN patients and HCs. H: human subjects constituting the pool, P: pool of human subjects. AN: anorexia nervosa, HC: healthy control.

Calculations were performed with R 3.5 software and GraphPad Prism software (version 7.00, La Jolla, CA, USA).

## Results

### Microbiota transfer was stable over time and maintained significant differences between groups

The patients diagnosed with anorexia nervosa (AN) enrolled in the study had a mean age of 29.3 (SD = 2.1) years and presented a mean BMI of 12.1 (SD = 0.1); the subjects in the healthy control group (HC) had a mean age of 27.7 (SD = 4.5) years and a mean BMI of 20.7 (SD = 1.2). According to French and European guidelines^34^^,^^58^ at the time of sampling, patients in the AN group were severely undernourished with more than 50% weight loss from their fitness weight and a low BMI (<13 kg/m^2^) due to a quantitative and qualitative reduction in calorie intake. Dietary survey reported a mean of 33% lower total energy intake (AN = 1333 kcal/d (SD = 115.5); HC = 2033 kcal/d (SD = 404.1); *p* = 0.044), with a significant difference in carbohydrates intake (AN = 161.7 g/d (SD = 45.4); HC = 281.7 g/d (SD = 55.9); *p* = 0.045). The patient's present score on the Hospital Anxiety and depression scale (HAD-S) anxiet y ≥ 11, corresponding to a certain presence of anxiety syndrome, whereas HAD-S depression score is ≥11 only for one of them. Scores evaluated by the Beck depression inventory coincide with moderate depression symptoms for two, and severe depression symptoms for one of the AN's group. Regarding obsessive and compulsive symptoms measured by the Maudsley Obsessional Compulsive Inventory (MOCI),^36^ two subjects from the AN group present a mid-range intensity of symptoms. Social phobia was evaluated using the Liebowitz scale; all three subjects in the AN group presented a pronounced symptomatology regarding activities in social context (specially eating–item 3), and activites under the sight of another person (items 6−8−9−11). The Godin scale estimates the activity of subjects, and all are classified as active. Regarding intestinal health, the Bristol scale evaluation report indicates no constipation or diarrhea, and for two subjects in the AN's group, a Francis scale score corresponding to irritable bowel syndrome is observed (Table 1).

**Table 1.** Phenotypic characteristics of patients and healthy controls.

	Subjects with anorexia nervosa			Healthy controls		
	#1	#2	#3	#1ʹ	#2ʹ	#3ʹ
General information						
Age	32	27	29	33	22	28
Body mass index	12	12.1	12.2	19	21	22
Length of disease (y)	19	12	13			
Nutritional status						
Albumin	36	42.2	39.2			
Transthyretin	0.13	0.21	0.2			
Aspartate aminotransferase (AST)	385	204	47			
Alanine aminotransferase (ALT)	506	412	164			
Kcal/d	1400	1400	1200	2500	1800	1800
Protein (g)	60	45	55	90	50	45
Lipid (g)	60	40	50	85	60	75
Carbohydrates (g)	155	210	120	344	265	236
Psychiatric symptomatology						
BDI (depression)	29	38	28	0	2	2
HAD-anxiety	15	16	14	1	6	7
HAD-depression	9	13	10	0	2	0
MOCI-cleaning	2	3	4	1	2	1
MOCI-checking	2	4	2	4	0	0
MOCI-slowness	2	2	3	0	2	0
MOCI-doubting	2	3	6	1	1	2
MOCI-total	8 low	12 mid	15 mid	6 low	5 low	3 low
Liebowitz-anxiety	29	36	26	7	25	15
Liebowitz-evitement	39	42	40	24	27	33
Liebowitz-global score	68	78	66	31	52	48
EDI-boulima	0	6	2			
EDI-interoceptive awareness	10	18	4			
EDI-impulse control	4	5	2			
EDI-desire for thinness	6	4	2			
EDI-inefficiency	9	10	17			
EDI-body dissatisfaction	12	12	12			
EDI-social insecurity	6	7	5			
EDI-distrust of interpersonal relationships	3	8	4			
EDI-perfectionism	13	3	3			
EDI-fear of maturity	10	2	5			
EDI-asceticism	9	9	9			
EDI-total	82	84	65			
Godin exercise	31	65	21	27	20	28
Intestinal symptomatology						
Francis score	318	25	90	25	25	0
Bristol scale	4	4	4	2	3	3

Legend: presentation of symptomatology evaluated in groups using the usual evaluation by self and non-self-questionnaires.

The microbiota diversity and richness did not differ between AN and HC individuals (Wilcoxon test: Shannon-*p-*value = 0.19, Richness–*p-*value = 0.08; Figure 1A). As expected with the poor number of subjects per group (*n* = 3), microbiota dissimilarity analysis (Figure 1B) did not allow the discrimination of AN microbiota composition from that of HCs (PERMANOVA *p* = 0.238). The bacterial compositions of AN patients and HCs were similar at the family level. The fecal pools reflect each group's average GM composition accordingly (Figure 1C). Two ASVs, ASV492 and ASV468, which are close to the *Flavonifractor* and *Mediterraneibacter* genera, respectively, are highly abundant in the AN microbiota compared with HCs (Figure 1D); the statistics and taxonomy of these ASVs are available in the Additional Table file (Supplementary Table 1).

**Figure 2.**
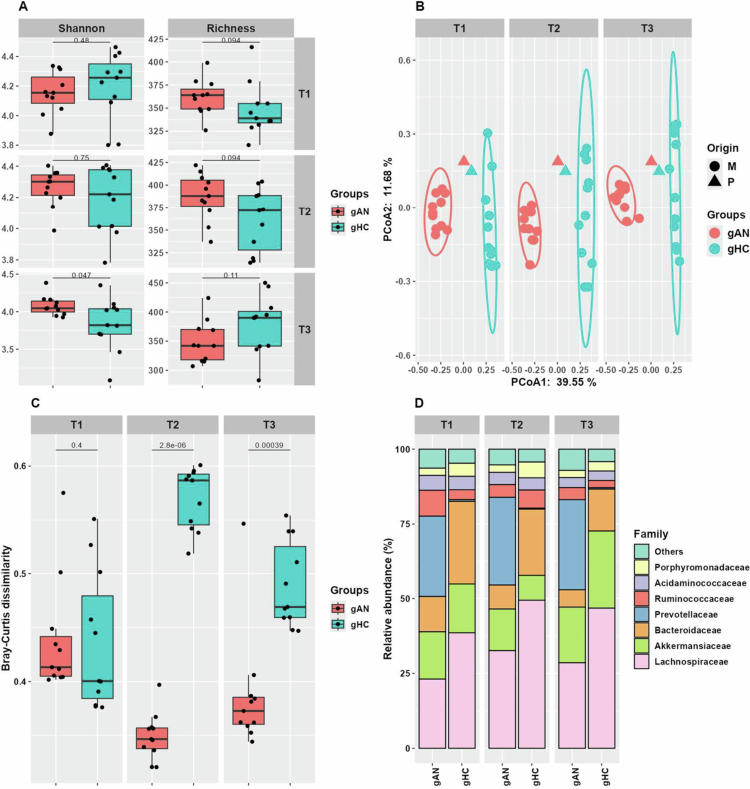
Analysis of the mouse microbiota composition in groups of humans and mice. A: Evolution of microbiota diversity and richness between groups at T1, T2, and T3 in mice receiving fecal microbiota transfer from ANs and HCs. B: Principal coordinate analysis (PCoA) displaying the mouse and initial human pools microbiota dissimilarity according to sampling time points. C: Boxplot depicting the average within-group dissimilarity (Bray–Curtis) in the gAN and gHC groups according to time. D: Relative abundance of bacterial families in groups of mice over time. T1: 3 weeks after inoculation; T2: 7 weeks after inoculation; T3: 11 weeks after inoculation. gAN: gnotobiotic mice from the anorexia nervosa group; gHC: gnotobiotic mice from the healthy control group.

Analyzes were conducted on 11 mice in each group, rather than the initially planned 12, due to the natural death of one mouse in each group during the experiment. The GM composition of mice receiving the AN fecal microbiota transfer pool (gAN) and those receiving the HC fecal microbiota transfer pool (gHC) was analyzed three times during the protocol. While the diversity and richness did not differ between the gAN and gHC groups at T1 and T2, the microbiota diversity increased in gAN compared with gHC at 54 d post-transplantation (T3) (*p* = 0.047) (Figure 2A). Richness remains similar within groups at T3. The GM dissimilarity analysis revealed a significant difference between the gAN and gHC groups in terms of their GM profiles (PERMANOVA *p* = 0.001) (Figure 2B). At T1, the within-group microbiota dissimilarity was similar between gHC and gAN. While the within-group dissimilarity decreased for gAN at T2 and T3 compared with that at T1, the opposite trend was observed for gHC (Figure 2C). The dissimilarity within the gAN group is significantly lower at T2 and T3 than that computed for the gHC. Compared with gHC, gAN harbors a different microbiota composition at the bacterial family level.

**Figure 3.**
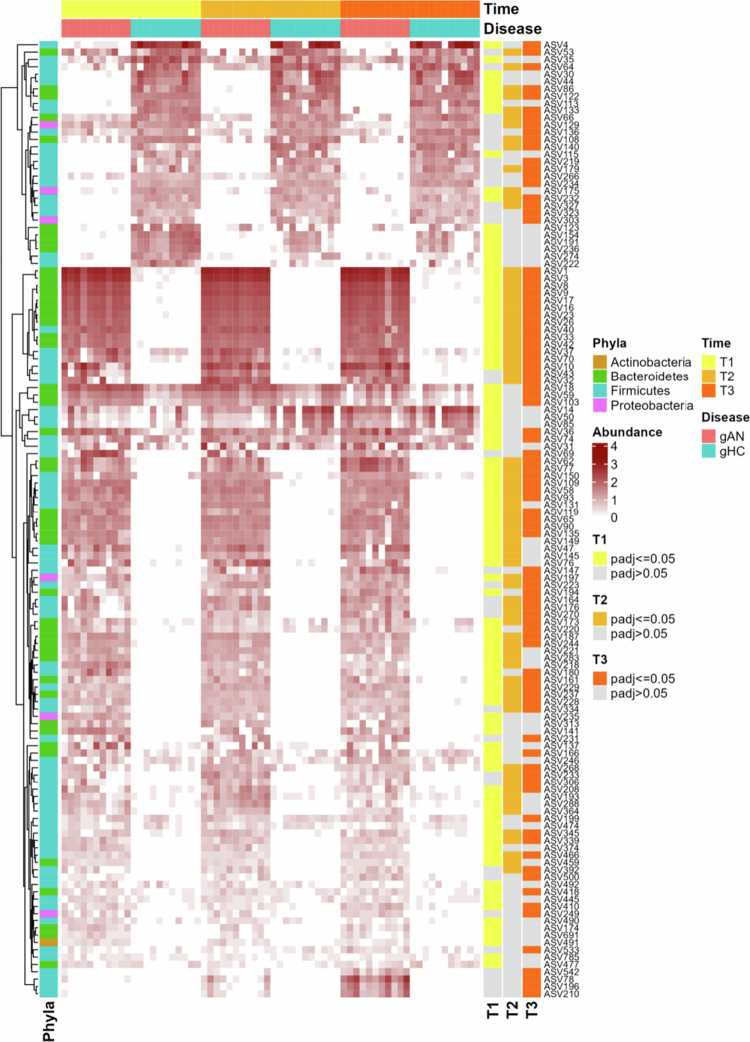
Microbiota features differentiate treatment groups. Corrected significance was set for a *p*-value = 0.05 for each time point. gAN: gnotobiotic mice from patients with anorexia nervosa, gHC: gnotobiotic mice from healthy controls. T1: 3 weeks after inoculation; T2: 7 weeks after inoculation; T3: 11 weeks after inoculation.

This observation is even more evident at the AVS level, with 131 ASVs whose abundance significantly shifted between gAN and gHC (Figure 3).

At T1, the relative abundance of 17 ASVs was increased in the gHC microbiota profile, and 80 ASVs were decreased compared with those in the gAN profile. Similar patterns were observed at T2 and T3, with an increased abundance of 21 and 19 ASVs, respectively, and a reduced abundance of 87 and 69 ASVs in gHC compared with gAN. Overall, the abundance of 41 ASVs significantly shifted at all time points between the gAN and gHC groups. The statistics and taxonomy of the ASVs are available in the Additional Table file (Supplementary Table 2).

**Figure 4.**
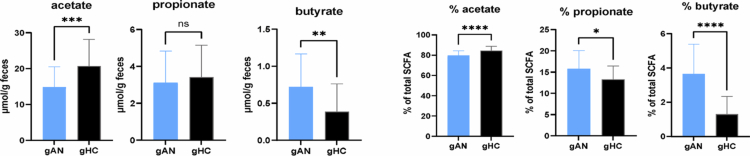
Short-chain fatty acid (SCFA) profiles in mice across different groups. Graphic representation of SCFA quantification from transplanted mouse feces expressed in µmol/g of feces and the percentage of total short-chain fatty acids. gAN: gnotobiotic mice from the anorexia nervosa group, gHC: gnotobiotic mice from the healthy control group. *: *p* = 0.05; **: *p *< 0.05; ***: *p *< 0.005; ****: *p *< 0.001; ns: not significant.

The short-chain fatty acid profile was assessed simultaneously with fecal microbiota analysis. The results revealed a significant difference in the acetate concentration in the stool (gAN = 14.89 µmol/g (SD = 5.68); gHC = 20.77 µmol/g (SD = 7.38); *p* = 0.0006) and in proportion to total SCFAs. The concentration of butyrate was also significantly elevated in the gAN fecal samples (gAM = 0.72 µmol/g (SD = 0.45); gHC = 0.39 µmol/g (SD = 0.37); *p* = 0.0019) and in the proportion of total SCFAs. The propionate concentration in gAN stool was significantly lower than the total SCFA concentration (gAN = 15.8% (SD = 4.28); gHC = 13.3% (SD = 3.1); *p* = 0.02) (Figure 4). Taking together, our results depict durable implantation of the two different microbiotas after transfer in each group, associated with a difference in the SCFA concentration in feces, suggesting a potential impact on host physiology.

### AN microbiota transfer induces lower food consumption and influences body composition

**Figure 5.**
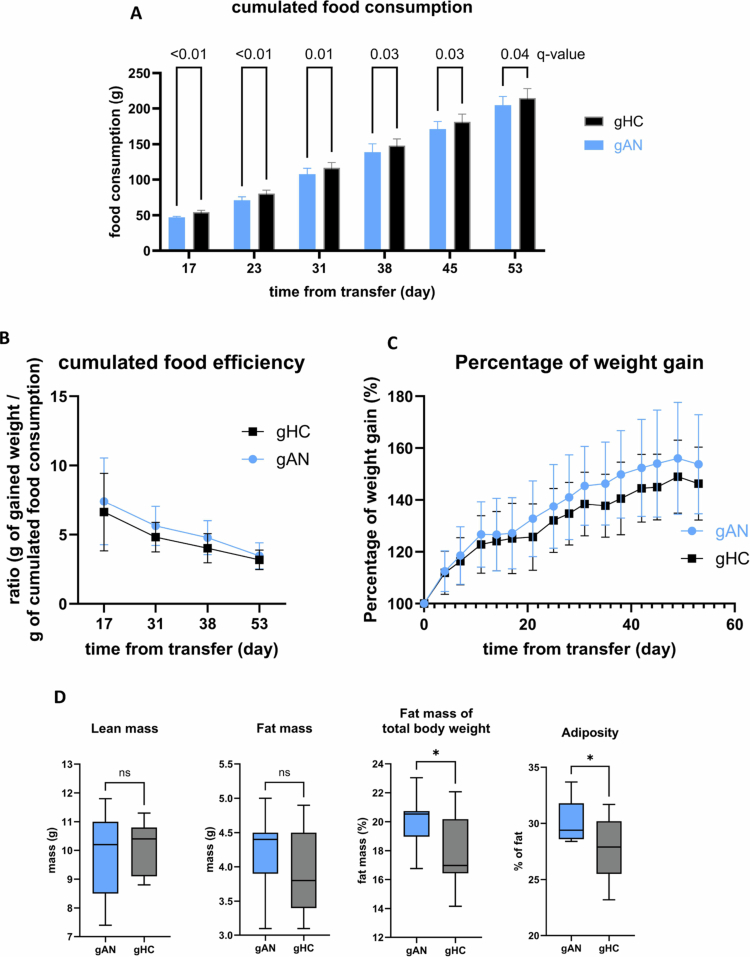
Graphical presentation of food consumption, weight, and body composition in groups. A: Cumulative food intake across categories. The significance of the mean comparison test is expressed as the q value (multiple Mann‒Whitney tests). B: Percentage of weight gained during the study. C: Food efficiency comparison between groups. Food efficiency was calculated by dividing the weight gain (in grams) by the cumulative food consumption (in grams). D: Graphic representation of the values (min, max, and median) of fat mass and lean mass measured via biphotonic absorptiometry and of the calculated adiposity and percentage of fat mass in total body weight at the end of the protocol in each group. gAN: gnotobiotic mice from the anorexia nervosa group; gHC: gnotobiotic mice from the healthy control group; *: *p* = 0.04.

**Figure 6.**
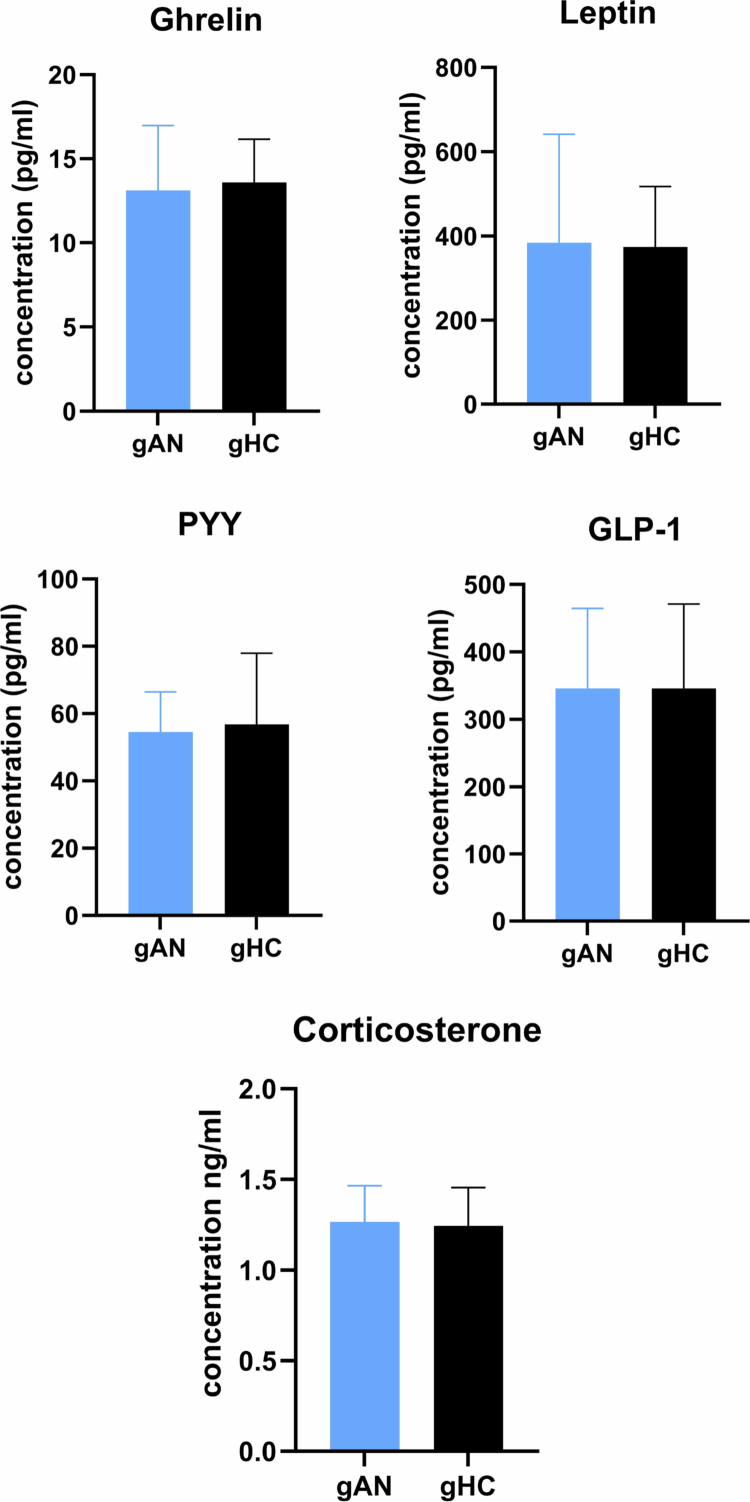
Hormone profiles in groups. The concentration of enterohormones in the serum was assayed via multiplex ELISA, and the corticosterone concentration was assayed via ELISA. The analysis did not reveal differences between groups. PYY: peptide YY; GLP1: glucagon-like peptide 1; gAN: gnotobiotic mice from the anorexia nervosa group; gHC: gnotobiotic mice from the healthy control group.

Reduced food intake is a primary characteristic of AN. The cumulative food intake was significantly lower in gAN mice than in gHC mice throughout the entire protocol (Figure 5A). The cumulative food efficiency, which represents weight gain per unit of consumed food, did not significantly differ between gAN and gHC after microbiota transfer (Figure 5B). Weight gain after microbiota transfer did not vary substantially between gAN and gHC (Figure 5C). The weight gain percentage during the protocol did not differ substantially (Supplementary Figure 1). Dual X-ray absorptiometry (Figure 5D) was used to measure the body's composition. Whereas bone mineral density, fat mass, and lean mass did not significantly differ between groups, there was a significant difference in adiposity (percentage of fat mass/percentage of lean mass) in favor of gAN (gAN: 30.0% (SD = 1.8%); gHC: 27.9% (SD = 2.7); *p* = 0.04). Additionally, the percentage of fat mass at the end of the protocol was significantly greater in gAN mice (20.10% (SD = 1.7); *p* = 0.045) than in gHC mice (18.12%, (SD = 2.6)). The systemic concentration of appetite-regulating hormones is known to be perturbed in patients suffering from AN. In our study, the blood serum concentrations of appetite-regulating hormones (ghrelin, leptin, PYY, and GLP-1) did not differ between the gAN and gHC groups (Figure 6). Additionally, the serum corticosterone levels were not significantly different between the groups (Figure 6).

Interestingly, the results concerning body composition in mice evoke the manifestations encountered in AN patients during nutritional care in cases of severe undernutrition. This may be related to the specificity of microbiota from patients, which could present better abilities to extract nutrients and produce energetic substrate. This could be related to the higher production of SCFAs in the gAN group, as exposed previously.

### The AN microbiota impacts physical activity and anxiety-like behavior in mice

Behavioral symptoms are a key feature of AN outcomes. Non-adapted physical activity is a clinical behavior characteristic of patients with AN.

**Figure 7.**
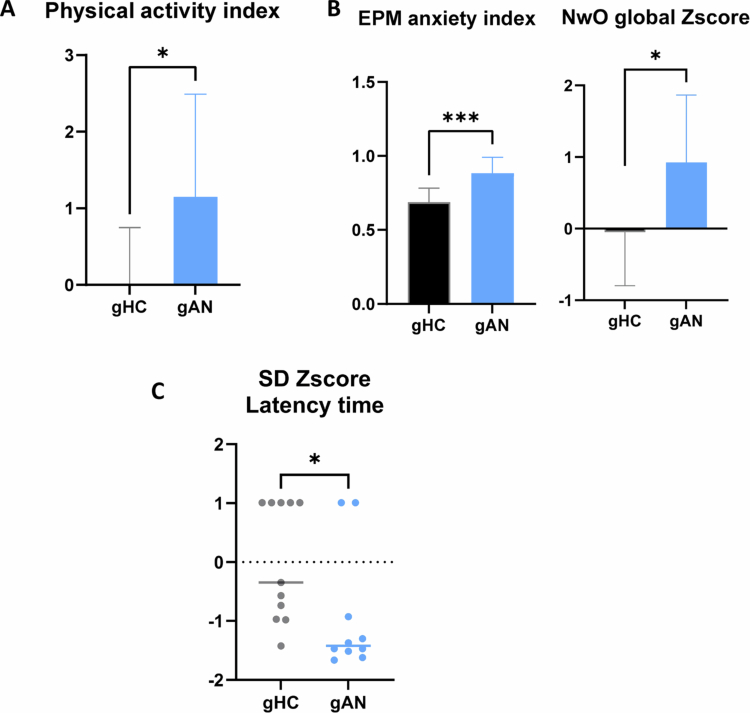
Significant behavioral test results regarding groups. A: The physical activity index was calculated from the average Z-score from the distance traveled, the number of rearings, and the speed measured in the open field and novelty object tests. B: The anxiety index was calculated from the number of entries and time spent in the open arms, divided by the number of entries and the total time spent in all arms. The novelty object (NwO) global Z-score is calculated by combining the mean visit time and latency to the first visit during the NO test. C: The latency to step down in the step-down (SD) test is represented as a Z-score considering the gHC group as a reference. gAN: gnotobiotic mice from the anorexia nervosa group, gHC: gnotobiotic mice from the healthy control group. *: *p *< 0.05.

We evaluated the physical activity of the mice via the open field (OF) test and the novelty object (NwO) test (detailed in Supplementary Figure 2). From dimensions of rearing count, distance travel, and number of entries in the central zone in the open field test, we calculated a combined Z-score of physical activity. The gAN mice displayed a greater physical activity index than the gHC mice did (gAN = 1.15 (SD = 1.338), gHC = 0.01 (SD = 0.733); *p* = 0.02) (Figure 7A). Significant difference in locomotion dimension in open field test does not allow to interpret dimension of number of entries in the center of the open field for its anxiety dimension.

Anxiety is a prevalent comorbidity in AN. We focus our effort on the anxiety index calculated from the elevated plus maze (EPM) test dimensions (Additional Mat & Met file and Supplementary Figure 3). Results revealed a greater level of anxiety in gAN mice than in gHC (*p* = 0.0003) (Figure 7B). In the novelty object (NwO) test, the global Z-score (composed of the mean visit time Z-score (*p* = 0.0014) and latency to first visit Z-score (ns) combination, details presented in (Supplementary Figure 3) was significantly different (*p* = 0.01), with interpretation of a greater level of anxiety in the gAN group (Figure 7B). The results of the step-down test are interpreted as a more anxious state in the case of higher latency for stepping down. Interestingly, the latency to step down in the so-called test was significantly greater in gHC (*p* = 0.02) (Figure 7C). We interpret the behavior of mice from the gAN group as substantially more impulsive than the behavior of mice in the gHC group. This statement is based on results showing a reduced time to step down for gAN mice, associated with a tendency for higher distance traveled and speed, as well as a higher number of entries in the center and rearing during the tests (Supplementary Figure 2).

As patients with AN frequently present depressive symptoms, tests evaluating the resignation dimension (apathy for the splash test and despair for the forced swim test) were carried out. The Z-score did not differ significantly in the splash test (SP) for grooming time or latency to first grooming, or in the forced swim test (FST) for latency to the first move or for immobility (Supplementary Figure 4). The anhedonia dimension remains to be explored.

Overall, these results align closely with the behavioral characteristics known to be symptoms in patients with AN. These include exacerbation of problematic physical activity and anxiety. Our results are directly associated with the specific transfer of intestinal microbiota from patients with AN.

### AN microbiota transfer in germ-free mice shapes the immune system

**Figure 8.**
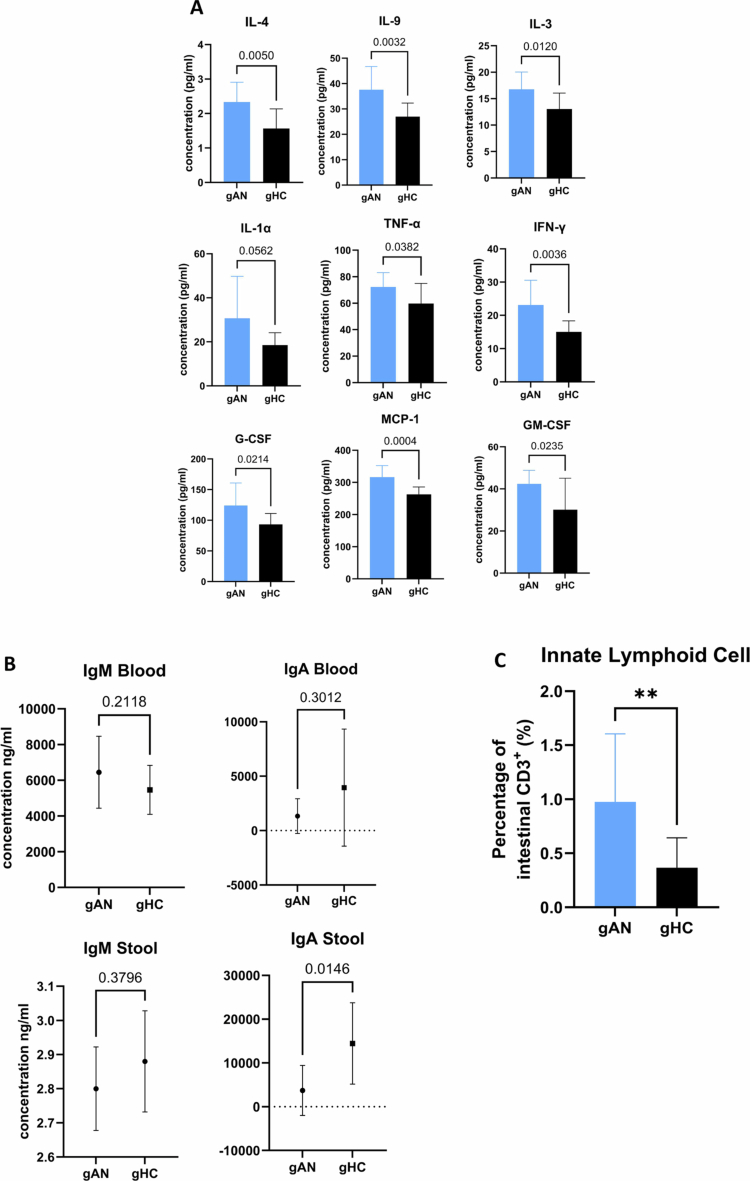
Immunological exploration reveals significant differences in cytokine concentrations and cellular counts. A: Cytokine concentrations were assayed by multiplex analysis in the serum of mice. Graphical presentation of comparisons of the mean concentrations, calculated via standard references, between groups for analytes with significant differences. B: Results from biological assays of immunoglobulin A and G concentrations measured in serum and stool via ELISA. C: Percentage of innate lymphoid cells measured via FACS as the percentage of CD3^+^ cells isolated from a cell mixture of intestinal mucosa samples. gAN: gnotobiotic mice from the anorexia nervosa group, gHC: gnotobiotic mice from the healthy control group. **: *p* = 0.005.

We evaluated immune responses by assaying circulating cytokines in mouse serum. Twenty-three cytokine concentrations were measured via multiplex analysis (Luminex technology). The mean concentrations were significantly greater in the gAN serum group than in the gHC serum group for eight analytes (*p *< 0.05), and there was a trend (*p* = 0.056) for one analyte (Figure 8A and Additional Table file Supplementary Table 3). Unfortunately, the IL-6 measure reports all samples as being below the detection range, which prevents calculation of the concentration. Elevated levels of IL-4 and IL-9 suggest a Th2 profile of the inflammatory response associated with allergy and IL-13 response. Elevated IL-3, IFN-*γ*, MCP-1, and GM-CSF promote the immune response to the macrophage response. Higher levels of IFN-*γ*, TNF-*α*, and G-CSF indicate a general inflammatory response. This inflammatory response could be secondary to fecal microbiota transfer and the implantation of a particular microbiome that triggers the immune system to elicit a long-lasting response polarized towards Th2. The results for the other analytes are provided in the Sup. Figure 5. Secretory immunoglobulins are essential mediators of host–microbiota symbiosis. The concentrations of IgA were lower in the feces of the gAN group (gAN: 3726 ng/ml (SD = 5714); gHC: 14,461 ng/ml (SD = 9289); *p* = 0.015). The concentrations of IgA in the serum and IgM in the serum and feces did not differ significantly between the groups (Figure 8B). These results suggest that mucosal immunity is less effectively reconstituted when microbiota from patients with AN are transplanted. We analyzed the frequency of lymphocyte populations in blood and intestinal mucosa samples to complete the immunological phenotyping. Canonical populations were present in both groups without significant differences (Supplementary Figure 6). Interestingly, a significant difference in innate lymphoid cells, which are implicated in the barrier function of the mucosa, was detected, with a lower frequency in intestinal samples from gAN mice than in those from gHC mice (Figure 8C). These results support a mucosal reaction toward the microbiota from patients with AN, suggesting the disruption of the immune route of the microbiota‒gut‒brain axis.

### AN microbiota transfer impacts organs beyond the gut

**Figure 9.**
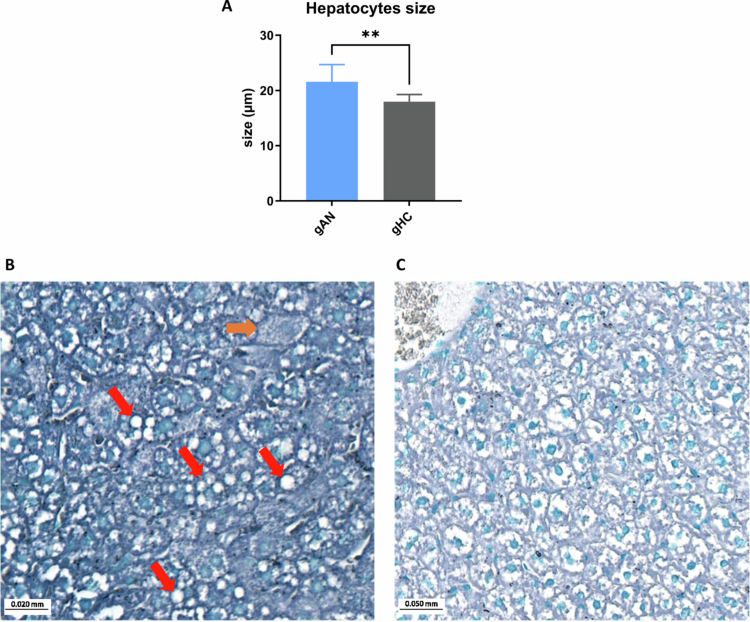
Hepatocytes' size difference between groups. Comparison of hepatocyte size measured from hepatic sections between groups. gAN: gnotobiotic mice from the anorexia nervosa group, gHC: gnotobiotic mice from the healthy control group. **: *p *< 0.005; B: hepatic section colored by Black Sudan B and methyl green. Orange arrows show ballooned cells. Red arrows show microvacuole of steatosis. C: Hepatic section presenting an empty cell interpreted as glycogenic overload, remaining empty secondary to solvent dissolution of glycogen.

The physiological complications of AN affect the liver and ovarian tissues.^59^^,^^60^ At the time of euthanasia, two mice in the gAN group presented macroscopic hepatic steatosis. A histological study revealed hepatocyte size was significantly greater (*p* = 0.0013) in the gAN group (21.6 µm, SD = 3.1) than in the gHC group (18 µm, SD = 1.3) (Figure 9A). The presence of ballooned hepatocytes in slices indicating steatosis in the gAN group (Figure 9B) and an abundance of hepatocytes with empty cytoplasm, which could be interpreted as a glycogenic overload (Figure 9C).

**Figure 10.**
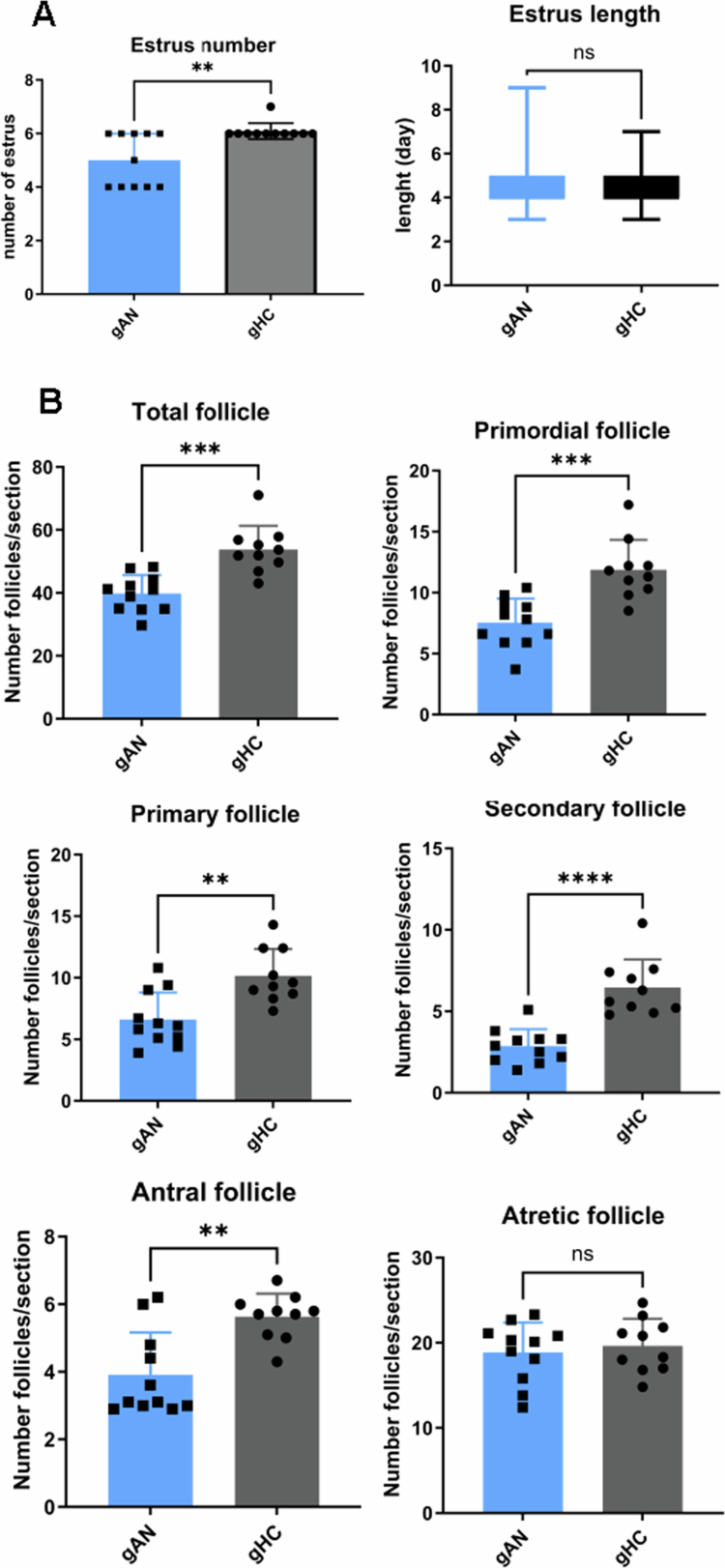
Estrus and ovarian histological differences in groups. Graphical representation of ovarian physiology in transplanted mice. A: Number of estrus cycles after transplantation (36 d) and comparison of the mean duration of estrus. B: Different follicles in each phase of ovarian development were counted in ovarian sections. gAN: gnotobiotic mice from the anorexia nervosa group, gHC: gnotobiotic mice from the healthy control group. **: *p *< 0.05; ***: *p *< 0.005; ****: *p *< 0.001.

Concerning the ovaries, the number of estrus cycles from weeks 4 to 9 differed significantly between the groups (Figure 10A), with fewer cycles in gAN mice (*p* = 0.009). The estrous cycle length, typically 4−5 d,^56^ did not differ substantially between the groups and was observed in the gAN group, with high variability (coefficient of variation gAN = 32.4%, whereas gHC = 19.9%). It is interpreted as an important irregularity in the cycle length. Histological analysis of the ovaries revealed important differences. The total number of follicles was significantly lower in the gAN group than in the gHC group. Significant differences were detected at all stages of follicle development, including primordial, primary, secondary, and antral follicles (Figure 10B). The number of atretic follicles did not differ significantly between the groups, suggesting a blockade of follicular development in gAN, possibly due to systemic inflammation or microbiological dysbiosis.

### Bacteria are associated with the manifestation of AN in gnotobiotic mice

**Figure 11.**
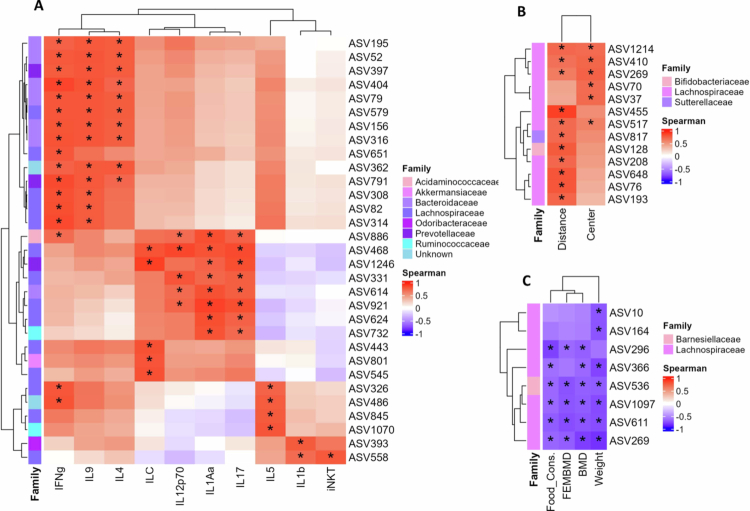
Correlation matrix between ASVs and biological parameters. Spearman correlation analysis results between amplicon sequence variants identified by 16S RNA gene metagenomic analysis and physiology assay results. A: Spearman correlation analysis of the following physiological parameters: food consumption (Food_Cons), femoral bone mineral density (FEMBMD), bone mineral density (BMD), and weight. B: Significant results of Spearman correlation analysis of immunological parameters. Interleukin (IL), interferon gamma (IFN-g), innate lymphoid cell (ILC), innate natural killer T lymphocyte (iNKT) C: significant results of Spearman correlation for the behavioral parameters of distance traveled and the number of center crosses in the open field test. *: *p*-value ≤ 0.01, absolut spearman |*ρ*| ≥ 0.5.

To determine whether the microbiological specificities of the microbiota from patients with AN can explain the observed manifestations in our study, we performed a correlation analysis between ASVs and the phenotyping parameters of the gAN group. The covariation results are reported graphically (Figure 11). Most positive correlations are associated with the family Lachnospiraceae. This family of bacteria belongs to the Firmicutes phylum and comprises most gut tract bacteria, characterized by their anaerobic and fermentative properties. These bacteria are associated with health conditions due to the abilities of SCFA (butyrate). In disease conditions, this family is related to metabolic diseases (obesity, diabetes), and is sensitive to diet variation.^61^ Positive correlations are identified with immunologic parameters identified in mice from AN group: mostly pro-inflammatory: IL-1, IL-17, and IL-12, also with the ILC count in gut tissue (Figure 11A). Regarding behavioral parameters, distance travel, one component of the hyperactivity identified positively correlates with the Lachnospiraceae (Figure 11B). This family is also recognized as negatively associated with bone mineral density, weight and food consumption (Figure 11C). A recent study about patients suffering from anorexia nervosa identified a potential causal role for the Lachnospiraceae.^62^ Interestingly, the immunologic parameters of IFN-*γ*, IL-4, and IL-9, which are involved in Th2 inflammatory polarization, are positively associated with the Bacteroidaceae, including SCFA-producing (acetate and propionate) strains, and in the metabolism of biliary acids. These correlations identified ASVs significantly associated with parameters depicting the phenotype of the gAN group, reinforcing the idea that the microbiota is involved in the pathophysiology of AN.

## Discussion

Our results support the hypothesis that transferring gut microbiota from donors with severe AN induces canonical features in a germ-free mouse model. These findings reinforce the evidence of a causal link between GM and AN symptoms, as described in patients. Transferred GM comes from severely female patients suffering from long-lasting AN who are recruited in a clinical nutrition department and who are performing nutritional rehabilitation of extremely undernourished patients. We chose patients with chronic and severe forms of AN to optimize homogeneity between disease state and GM.

In our study, gnotobiotic mice from the gAN group exhibited significantly lower cumulative food intake, even when food was available ad libitum. These results are in line with those of Hata et al.^31^ This difference in food consumption is not linked to the disruption of enterohormone concentrations, which are similar between groups of mice at the end of the protocol. This statement needs to be reproduced and specified, as our assays were performed at the end of our protocol (8 weeks) and did not evaluate the evolution of hormones under specific conditions for sampling and analysis of enterohormones. This condition contrasts with the known characteristics in humans of elevated ghrelin, PYY, and cortisol concentrations, and low concentrations of leptin and GLP−1, as it is associated with chronic food deprivation and chronic undernutrition.^63^^,^^64^ The quantitative assessment of food consumption in subjects with AN was also halved compared to the HC group. Despite similar weight gains in the two groups, the body composition of the mice in the gAN group differed, with greater fat mass in the gAN group than in the gHC group, even though the diet and housing conditions were similar. The fat gain percentage was also significantly increased after fecal transfer from patients with AN to mice in a recent study.^19^ This strongly suggests that AN dysbiosis is associated with fat mass gain despite the lack of difference in food efficiency between the groups. Fat mass gain after fasting is known as plastic in mice^65^ and appears to be dependent on hormonal status (IGF-1).^66^ Indeed, host IGF-1 production regulation is known to be inducible through the influence of the GM.^67^ Further studies need to consider plasma IGF-1 levels in gAN mice. In humans suffering from AN, this fat mass gain is a known effect of nutritional rehabilitation in patients treated during the acute phase of undernutrition^68^^,^^69^ predominantly in the trunk zone^70^ and linked to high cortisol levels associated with chronic starvation.^71^ Our results may be explained by the fact that the animals were not undernourished (under ad libitum feeding conditions). Indeed, the most common unbalanced diet in patients with AN is characterized by an enrichment in complex fiber from vegetables, with an imprecise proportion of soluble and insoluble fiber, and a low intake of carbohydrates and protein.^72^ In our group of patients, only the intake of carbohydrates appears significantly reduced in patients suffering from AN. Feeding the mice a standard chow diet may explain our results, thereby modeling a refeeding condition with a balanced diet rather than the maintenance state of a chronic AN condition. As diet is a potent modulator of the microbiota,^73^ future microbiota transfer studies should involve a variety of different diets, mimicking caloric restriction and high-fiber intake, to investigate the maintenance of symptomatic manifestations in mice associated with symptoms of AN in humans.

gAN Mice presented a significant increase in physical activity, associated with an interpretation of impulsivity, which models the problematic physical activity observed in patients with AN. Scores reported in the three patients from the AN group from Godin's scale corresponded to the active state. This statement is supported by interpreting composite Z-scores and considering results from multiple behavioral tests.^46^ Our results depicted a higher level of anxiety in the gAN group. High levels of anxiety symptoms have been reported in persons suffering from AN; these symptoms are at least partially linked to malnutrition in multiple investigations.^74^,​​​​​​^75^ A recent study described a positive relationship between the course of anxiety disorder syndrome and nutritional status during the inpatient treatment of AN; however, the underlying biological mechanism remains unclear. Some studies have established a link between GM dysbiosis and depressive symptoms.^20^ However, we did not identify apathy and despair behavioral characteristics in the gAN model mice, and anhedonia still needs to be explored. These results may be related to the measure of depression scale (BDI and HAD-S) used to estimate the presence of depressive symptoms in the patients of the AN group. Additionally, the absence of differences in depression scores may be due to the brief duration of our protocol. Individual housing conditions induce stress, but it is still less severe than the mild chronic stress model used for depression assessment.^76^ Maintaining gnotobiotic conditions for extended periods may provide insight into the kinetics of the onset of behavioral manifestations.

Biological results revealed an inflammatory profile associated with the Th2 lineage, an innate immune reaction, and a significantly greater number of innate lymphoid cells in intestine samples from the gAN group. The lack of IL-6 dosage is regrettable as this pro-inflammatory cytokine is associated with specific stages of anorexia nervosa in humans^23^^,​^​​​​​​^77^ but also remains controversial in longitudinal studies.^24,^^25^^,^^27^ We detected a significantly lower concentration of IgA in the stool between the groups. In the immune phenotyping assay, we detected the reconstitution of Peyer's patches in the intestine, which were visible during dissection, and the absence of differences in lymphocyte populations. Even the axenic condition of the animal could first explain this; we infer that IgA depletion is linked to the bacterial specificity of the microbiota from AN. Secretory IgA is essential for microbiota-host symbiosis, and a close relationship exists between immunoglobulins and the microbiota through antibody-mediated immune selection.^78^ Disruption of this physiological mechanism in AN may explain dysbiosis. Currently, no data exists on the fecal IgA concentration in patients with AN, and our team is conducting a study to address this gap (NCT05842343). Some previous studies in patients with AN reported an elevated ratio of CD4^+^/CD8^+^ lymphocytes;^79^ in contrast, chronic AN appears to be associated with an increase in regulatory T cells.^28^ Cellular immune deficiency is linked to human malnutrition, which is not the case for our model. The increased intestinal permeability may explain this systemic inflammation. Nevertheless, the results of the ELISA for zonulin in the stool remained below the detection limit for both groups, and the level of calprotectin did not significantly differ (gAN = 4.51 pg/mg, (SD = 2.66); gHC = 5.61 pg/mg, (SD = 7.35)). However, the evidence for intestinal permeability in patients with AN is inconsistent from one study to another.^80^ The immune status in different stages of AN remains unclear. It represents a rich source of biological markers implicated in disrupting microbiota‒host symbiosis.

We noted that biological cytolysis, hepatic overload, increased hepatocyte size, steatosis, and abnormal glycogen reserves are common liver complications in patients with AN,^59^ and are associated primarily with chronic undernutrition^81^ and nutritional rehabilitation.^59^ The donors were in acute and severe conditions of undernutrition and presented biological cytolysis. This suggests a link between an unbalanced diet, GM, and hepatic manifestations in our model. These results align with the symptoms observed in humans.^82^ Steatosis is encountered mainly during nutritional rehabilitation and may also depend on the specific microbiota of patients with AN.^83^

Undernourished female patients with AN present with a fertility disorder with primary or secondary amenorrhea, and these disturbances improve with nutritional care, with a return to a regular menstrual cycle at normal weight in most cases. However, fertility disorders persist in some patients.^84^ In gAN mice, we observed profound alterations in the ovarian cycle. The results revealed a reduced number of estrus cycles and a lower total follicle count, which were also observed across all development phases. We suggest that specific GM from patients with AN impacts follicular reserve and follicular development. Interestingly, recent studies in mice have established a link between the depletion of ovarian reserves and a proinflammatory stage, one related to aging^85^ and the other related to stress.^86^^,^^87^ Therefore, the depletion observed in gAN mice may be associated with the elevated levels of inflammatory cytokines observed in our assays. As suggested, alterations in ovarian function may depend not only on nutritional status but also on the gut microbiota through the existence of a microbiota‒gut‒ovary axis that mainly involves estrogen metabolism through a reduction in *β*-glucuronidase activity.^88^ It is also known that the microbiota impacts the host endocrine system by disrupting the hypothalamic‒pituitary sexual axis. Understanding the effects of estrogen and the microbiota on AN requires further experiments.^89^ Unfortunately, this protocol does not include sex hormone assays or the exploration of the vaginal flora, which are also essential factors for ovarian physiology.^90^ These data match the disturbance of sexual physiology found in patients with AN suffering from profound undernutrition, such as our donors. The impairment of sexual function is common in patients with AN.^91^ A recent study hypothesized that a low plasma concentration of anti-Müllerian hormone (AMH) explains the diminished ovarian reserve and reproductive issues that occur during adulthood. Instead, an unexpectedly elevated concentration of anti-Mullerian hormone was found in patients suffering from AN.^92^ Additionally, hypercortisolism associated with prolonged feeding restriction contributes to ovarian cycle disruption through interactions with the hypothalamic‒pituitary‒gonadal axis.^93^ The relationship between AN mechanisms and sexual outcomes requires further study. Only female mice were used for our protocol. As AN is also a male disease, future protocols must consider microbiota transfer from male patients to male mice to identify similar transfers of symptoms from AN.

After transplantation, both microbiotas showed implantation and stability during the study. The concentration of SCFAs in feces exhibits an interesting pattern, with increased concentrations of butyrate and propionate in gAN mice and a lower concentration of acetate compared to the gHC group. In human studies of AN, the concentration of SCFAs has also been reported to be modulated. Low concentrations of propionate^22^^,^^94^ butyrate^22^^,^^95^ and acetate^94^^,^^95^ as well as increased concentrations of valerate and branched-chain fatty acids, have been reported in anorexic patients. These results pertain to the acute undernutrition stage because they were obtained from a sample collected at the time of inpatient admission. After refeeding, patients present with increasing proportions of butyrate and propionate in total SCFAs, while acetate remains low.^96^ Our results are consistent with the conditions of refeeding. The animals were fed a chow diet with ad libitum access to food. The composition of the chow diet is optimal for mouse physiology. It does not share features with the canonical diet of patients with AN, which is composed mainly of fiber nutrients and protein in proportion to the intake. Additionally, the vegetable-enriched diet of patients with AN may have been selected for butyrate-producing bacterial strains that contribute to the perturbation of host-microbiota symbiosis. SCFAs are negatively correlated with various behaviors in mice, particularly anxiety.^97-99^ Additionally, these essential metabolites influence the hypothalamic-pituitary-ovarian axis and modulate reproductive ability.^100^ Correlation analysis revealed that the *Blautia* genus is negatively associated with cytokine concentrations (TNF-*α* and IL−3). This genus of anaerobic bacteria, found in the intestines of mammals, is reportedly related to adipose tissue homeostasis through the production of acetic acid and protection against intestinal mucosa inflammation by upregulating intestinal regulatory T cells.^101^ The genus *Anaerobutyricum* is a butyrate-producing strain associated with insulin sensitization in human studies^102^ and is negatively associated with weight gain. These correlations encourage the isolation of these genera and the performance of metagenomic analyzes to achieve a level of understanding of bacterial species function. Hopes are shifting toward microbiological modulation, with a clinical trial currently underway that compares the administration of a cocktail of probiotics (Lactobacillus and Bifidobacterium strains) and is expected to yield results in terms of weight gain, psychiatric symptoms, and gastrointestinal symptoms.

The specific gut microbiota in patients with AN along the gut‒brain axis seems to be involved in the pathophysiology of this severe and life-threatening eating disorder and could act as a symptom trigger. To learn about the biological mechanisms involved, the next step requires exploring modifications to brain structure and function, i.e., considering that the reward system that functions, which is already known to be impaired in patients.^103^ Our study aimed to demonstrate that altered host–microbiota symbiosis shapes the physiology of mice in multiple aspects after the standardized transfer of fecal microbiota from a chronic AN patient into an acute state of undernutrition. We propose a robust model of AN symptomatology, which is based on fecal microbiota transfer, to study the physiological modifications induced by the consequences of restrictive eating behavior disorders. This mouse model facilitates the generation of hypotheses for future clinical studies, offering a resolution that is unattainable in humans, and can be utilized to target the microbiota for phenotyping, prognosis, and treatment of AN.

Guarantor of the article: Mouna Hanachi; email: mouna.hanachi@aphp.fr

## Supplementary Material

Supplementary Material Supp_Figure_6.pdf

Supplementary Material Supplementary Material

Supplementary Material Supplementary Material

Supplementary Material Supplementary Material

Supplementary Material Supplementary Material

Supplementary Material Supplementary Material

Supplementary Material Supplementary Material

Supplementary Material Supplementary Material

Supplementary Material Supplementary Material

Supplementary Material Supplementary Material

Supplementary Material Supplementary Material

Supplementary Material Supplementary Material

Supplementary Material Supplementary Material

Supplementary Material Supplementary Material

Supplementary Material Supplementary Material

Supplementary Material Supplementary Material

Supplementary Material Supplementary Material

## Data Availability

All data generated or analyzed during this study are included in this published article [and its supplementary information files]. The sequencing data are available on the NIH Bioproject platform, recorded under the number PRJNA1075908, or can be accessed via the following link: http:///www.ncbi.nlm.nih.gov/bioproject/?term=PRJNA1075908. Raw data from the experiment are available for download from https://entrepot.recherche.data.gouv.fr/ or following this link: https://doi.org/10.57745/V7VEJM.
