# Supplementary material for: Anorexia nervosa symptoms are induced after specific gut microbiota dysbiosis transfer in germ-free mice
Source: Gut Microbes. 2025 Nov 15;17(1):2563701. doi: 10.1080/19490976.2025.2563701 (PMC12626428; doi:10.1080/19490976.2025.2563701)
Supplement: Supplementary Material — Supp_Figure_6.pdf [file KGMI_A_2563701_SM4981.pdf]

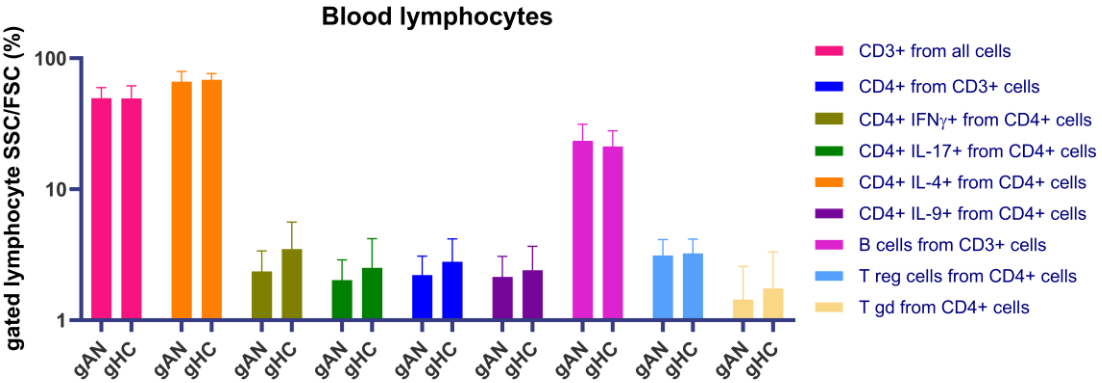

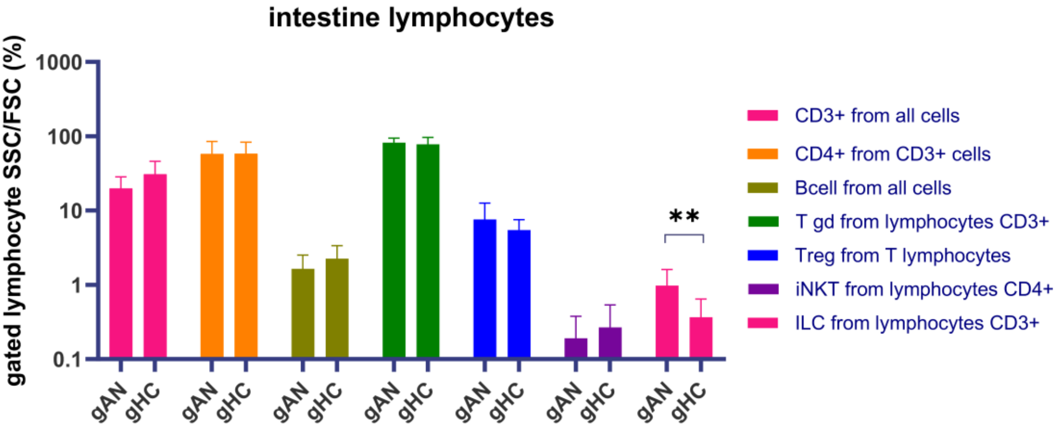

Supplementary Figure 4: detail of population identified using FACS. Labeling was performed in cells from whole blood and from an intestinal cell solution from an intestinal sample, which excluded Peyer's patches. gAN: gnotobiotic mice anorexia nervosa group, gHC: gnotobiotic mice healthy controls group, **: p=0,005
