# Supplementary figures and images for: Anorexia nervosa symptoms are induced after specific gut microbiota dysbiosis transfer in germ-free mice
Source: Gut Microbes. 2025 Nov 15;17(1):2563701. doi: 10.1080/19490976.2025.2563701 (PMC12626428; doi:10.1080/19490976.2025.2563701)

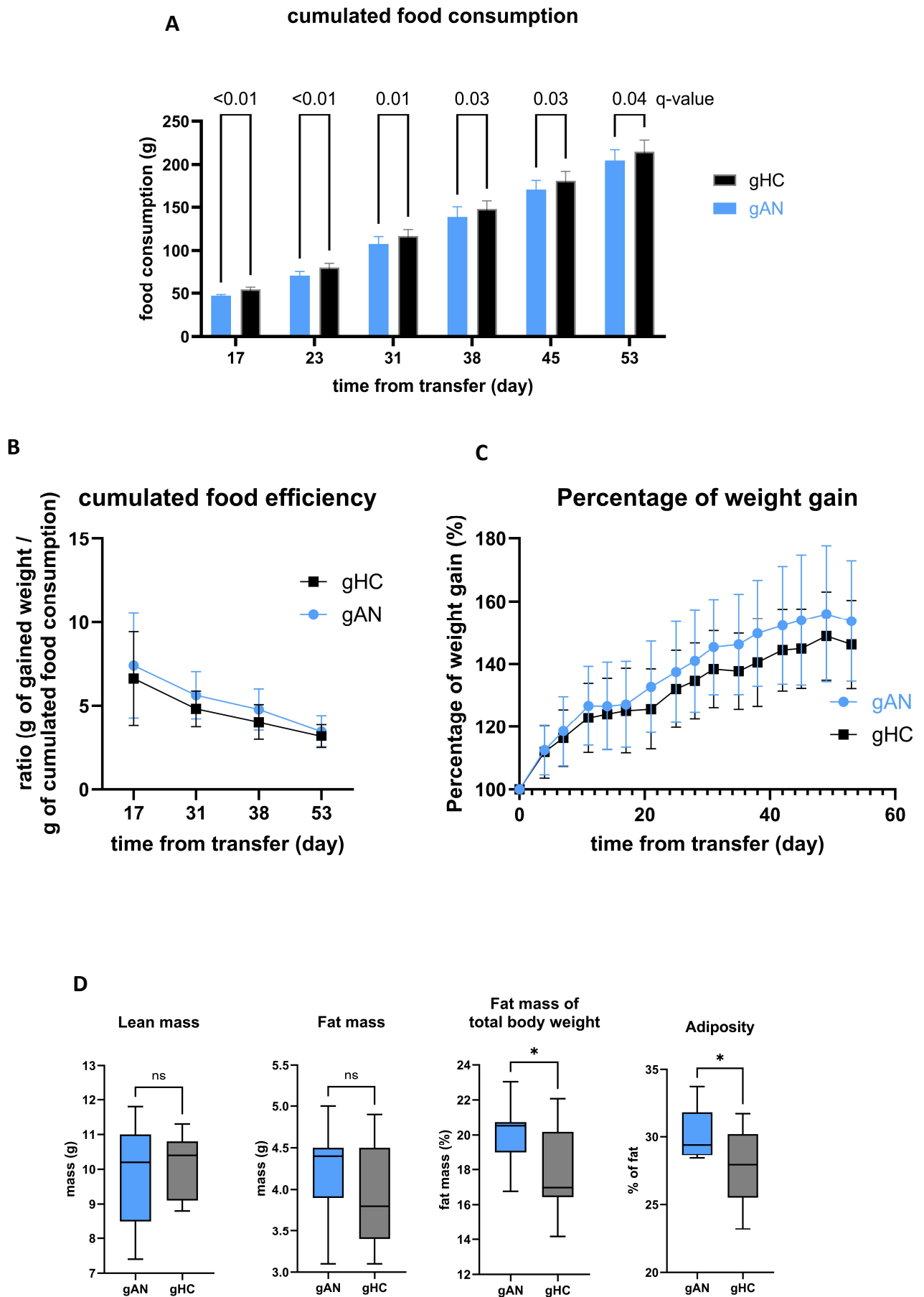

Gabriel-Segard et al. **Fig. 5**

Supplement: Supplementary Material [file KGMI_A_2563701_SM4980.pdf]

A

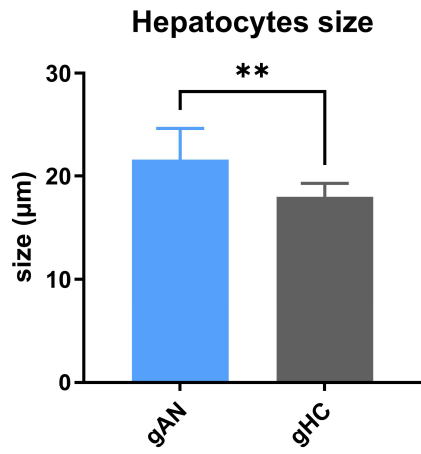

B

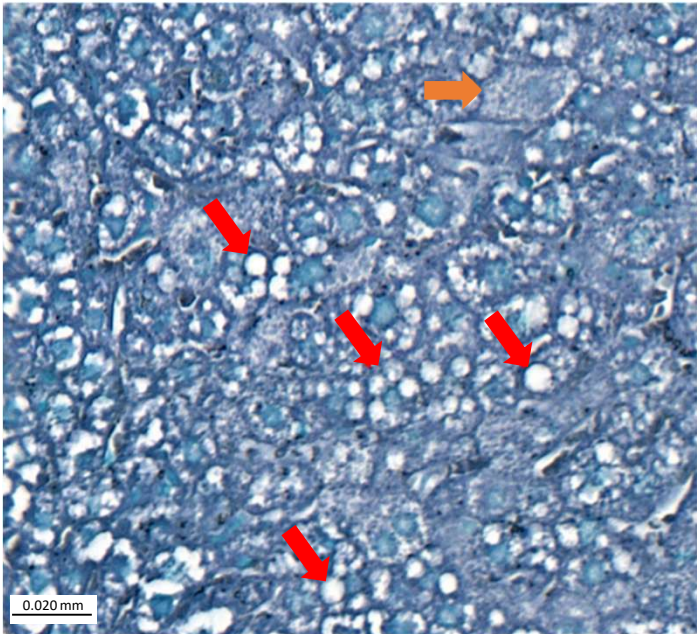

C

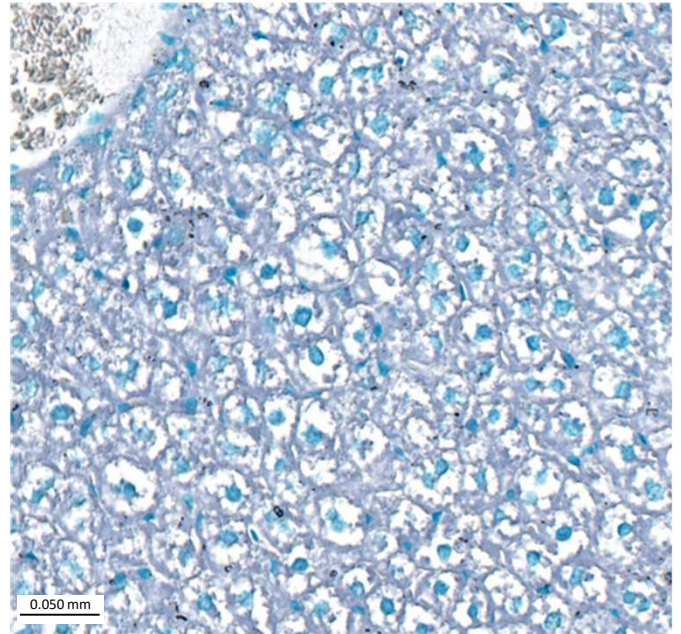

Supplement: Supplementary Material [file KGMI_A_2563701_SM4979.pdf]

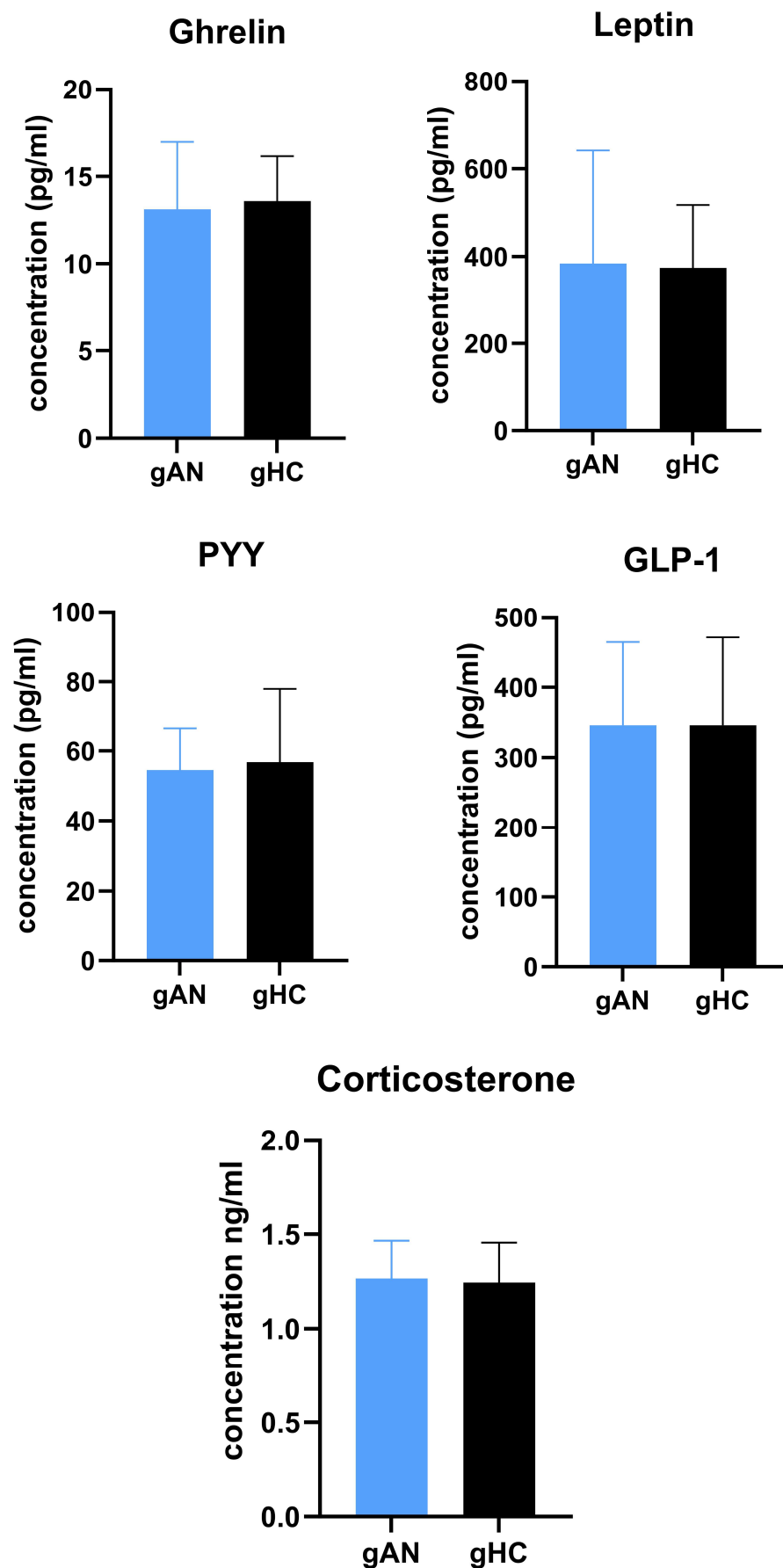

Gabriel-Segard et al. **Fig. 6**

Supplement: Supplementary Material [file KGMI_A_2563701_SM4978.pdf]

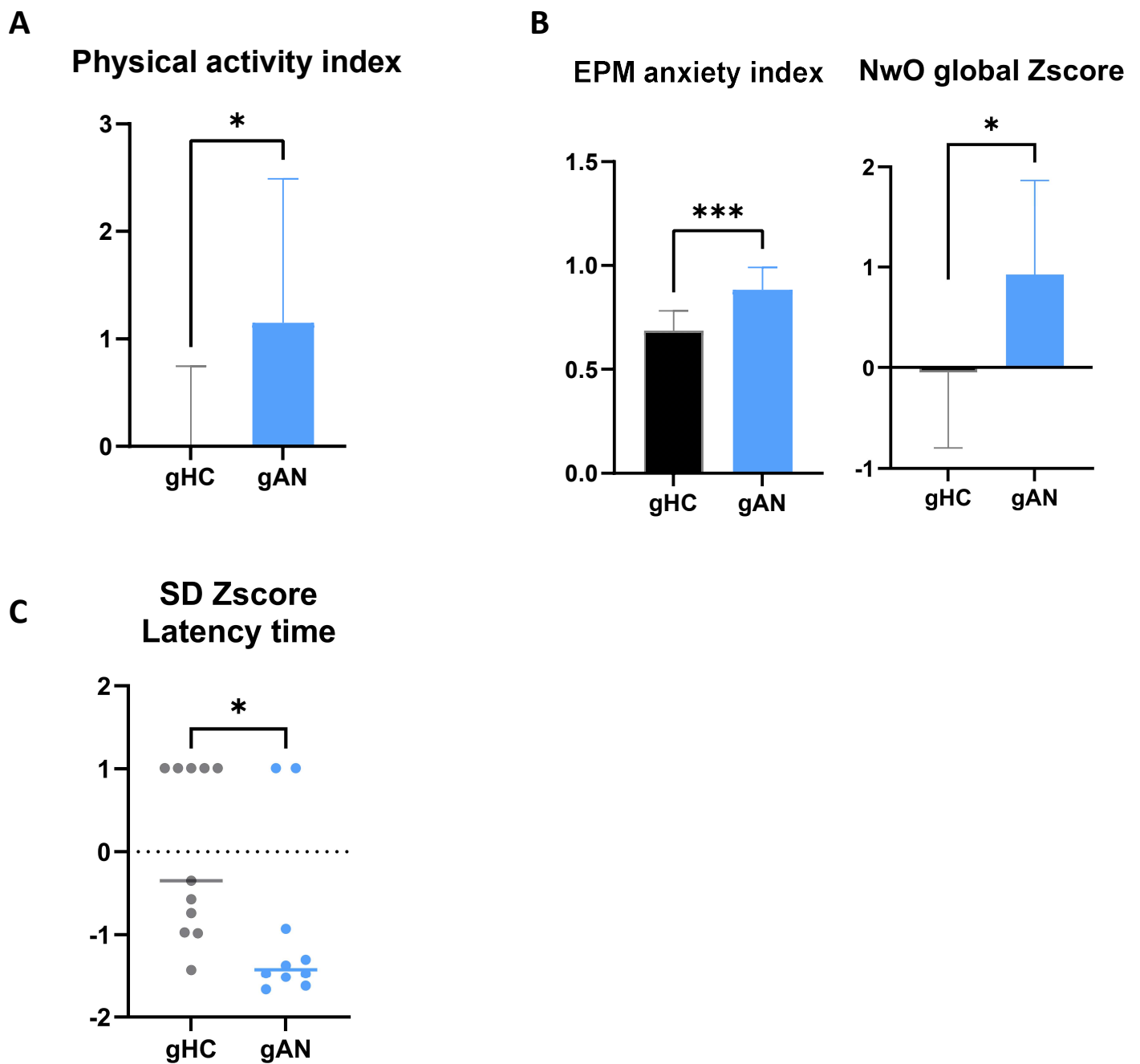

Supplement: Supplementary Material [file KGMI_A_2563701_SM4977.pdf]

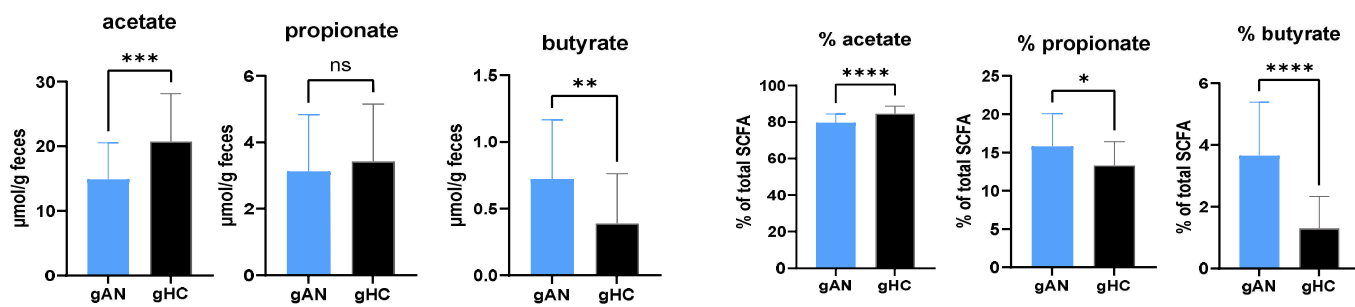

Supplement: Supplementary Material [file KGMI_A_2563701_SM4975.pdf]

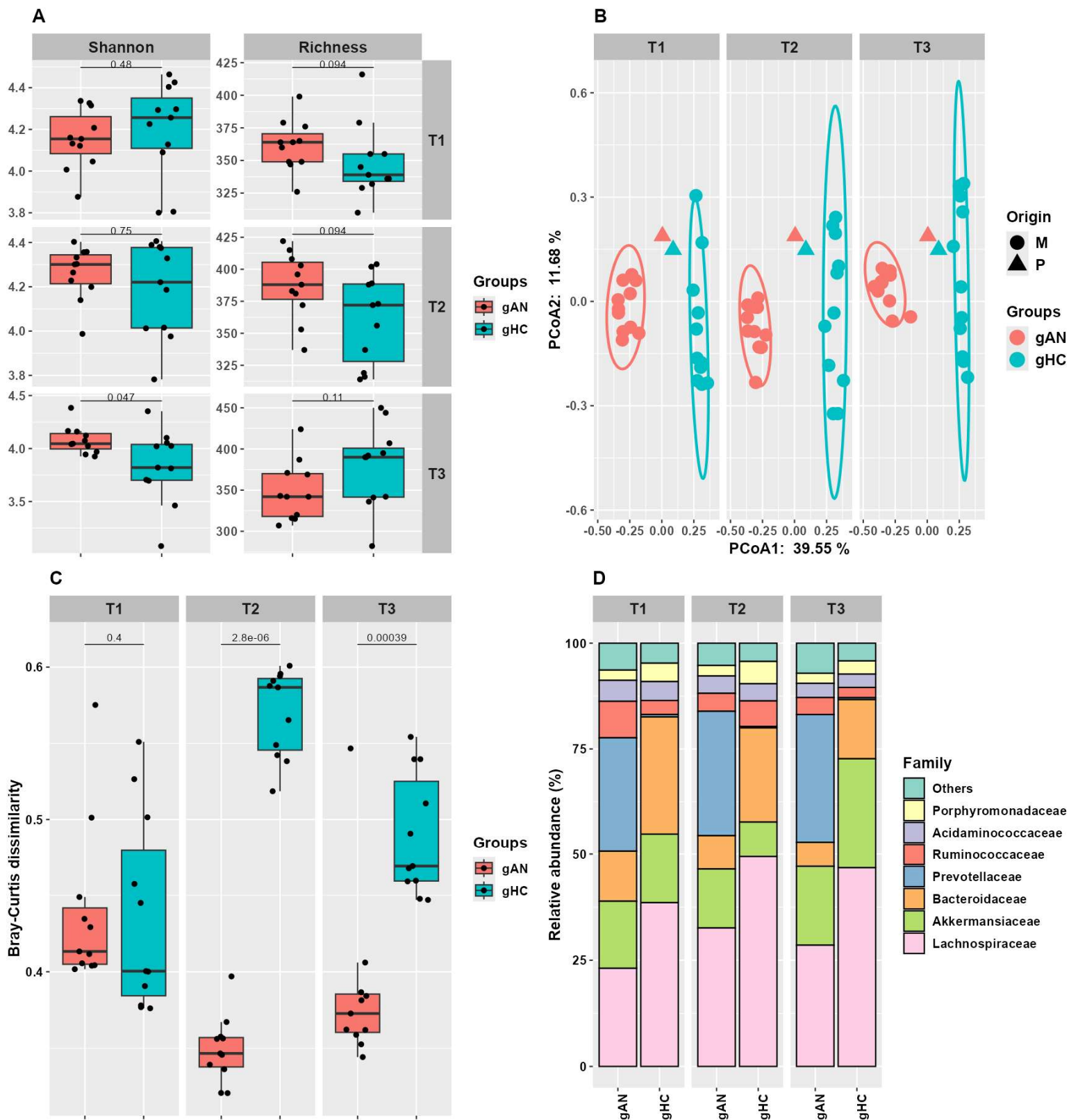

Gabriel-Segard et al. **Fig. 2**

Supplement: Supplementary Material [file KGMI_A_2563701_SM4973.pdf]

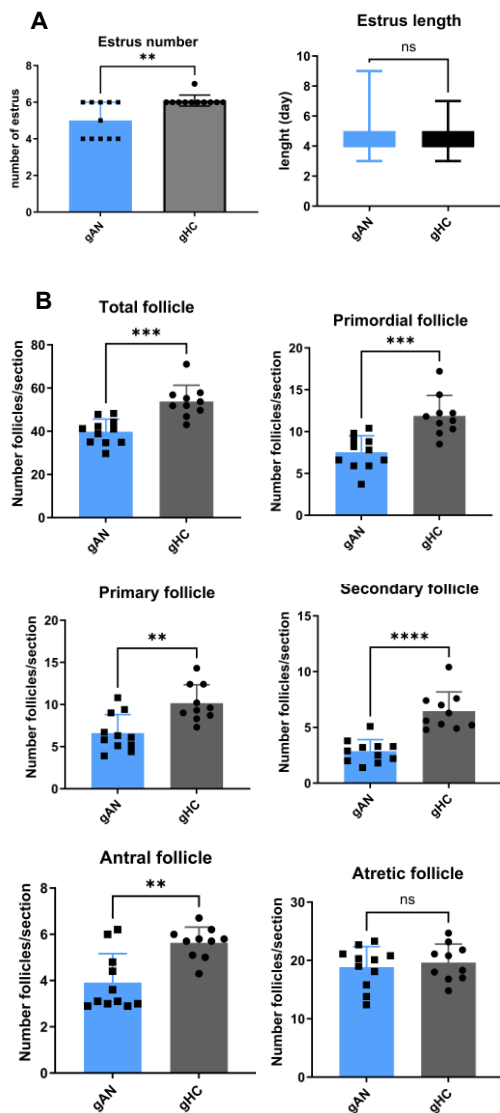

Supplement: Supplementary Material [file KGMI_A_2563701_SM4972.pdf]

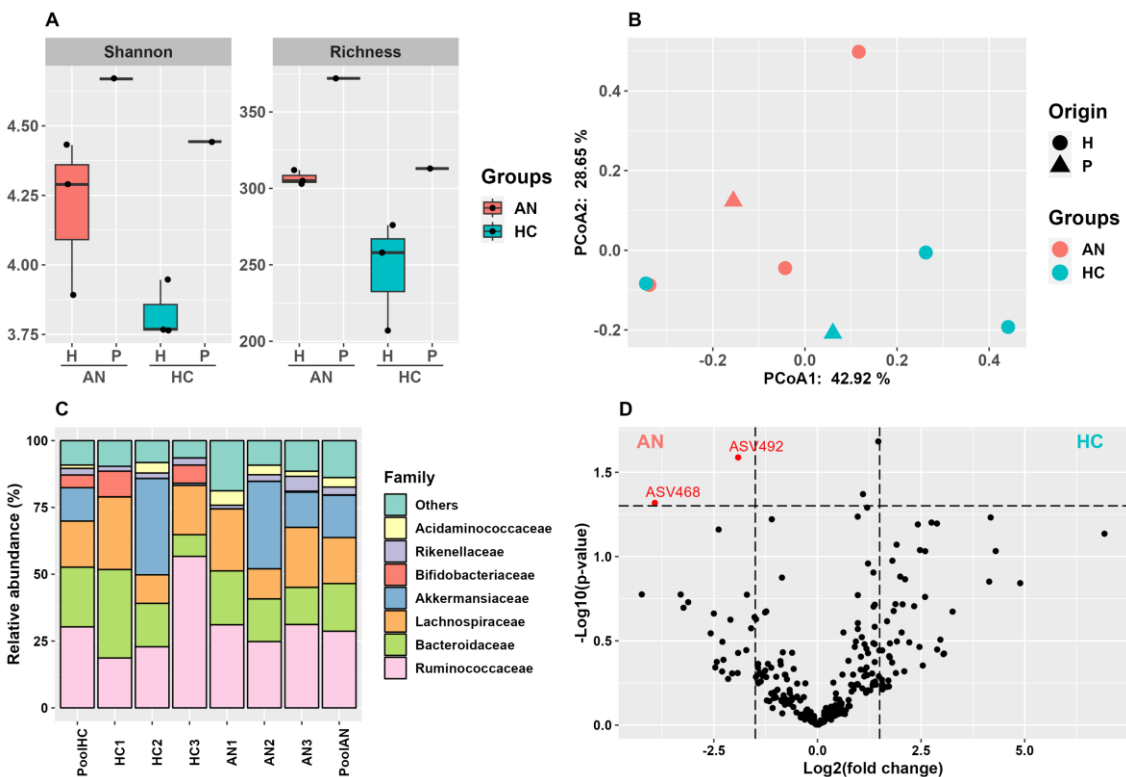

Gabriel-Segard et al. **Fig. 1**

Supplement: Supplementary Material [file KGMI_A_2563701_SM4970.pdf]

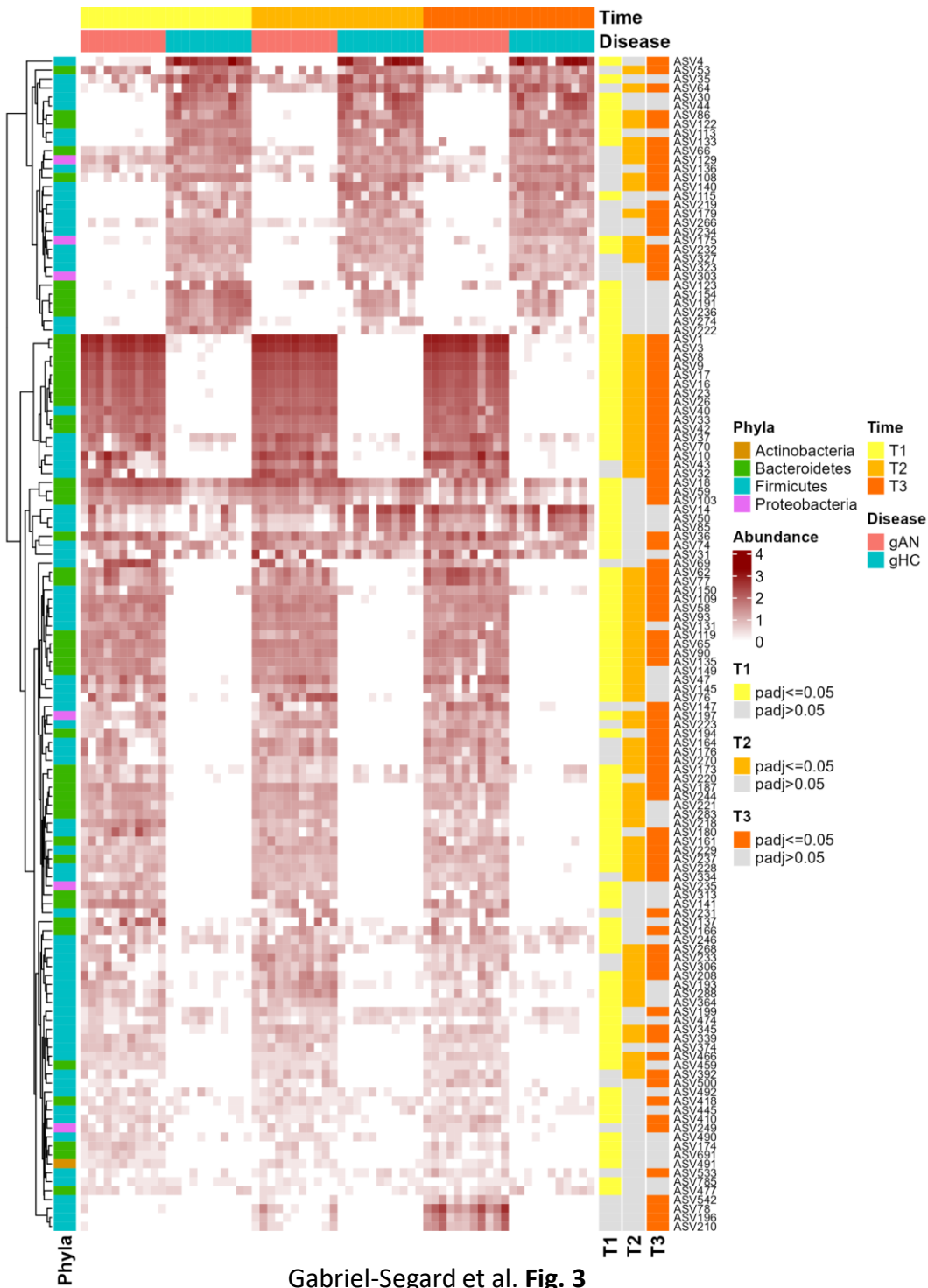

Gabriel-Segard et al. **Fig. 3**

Supplement: Supplementary Material [file KGMI_A_2563701_SM4968.pdf]

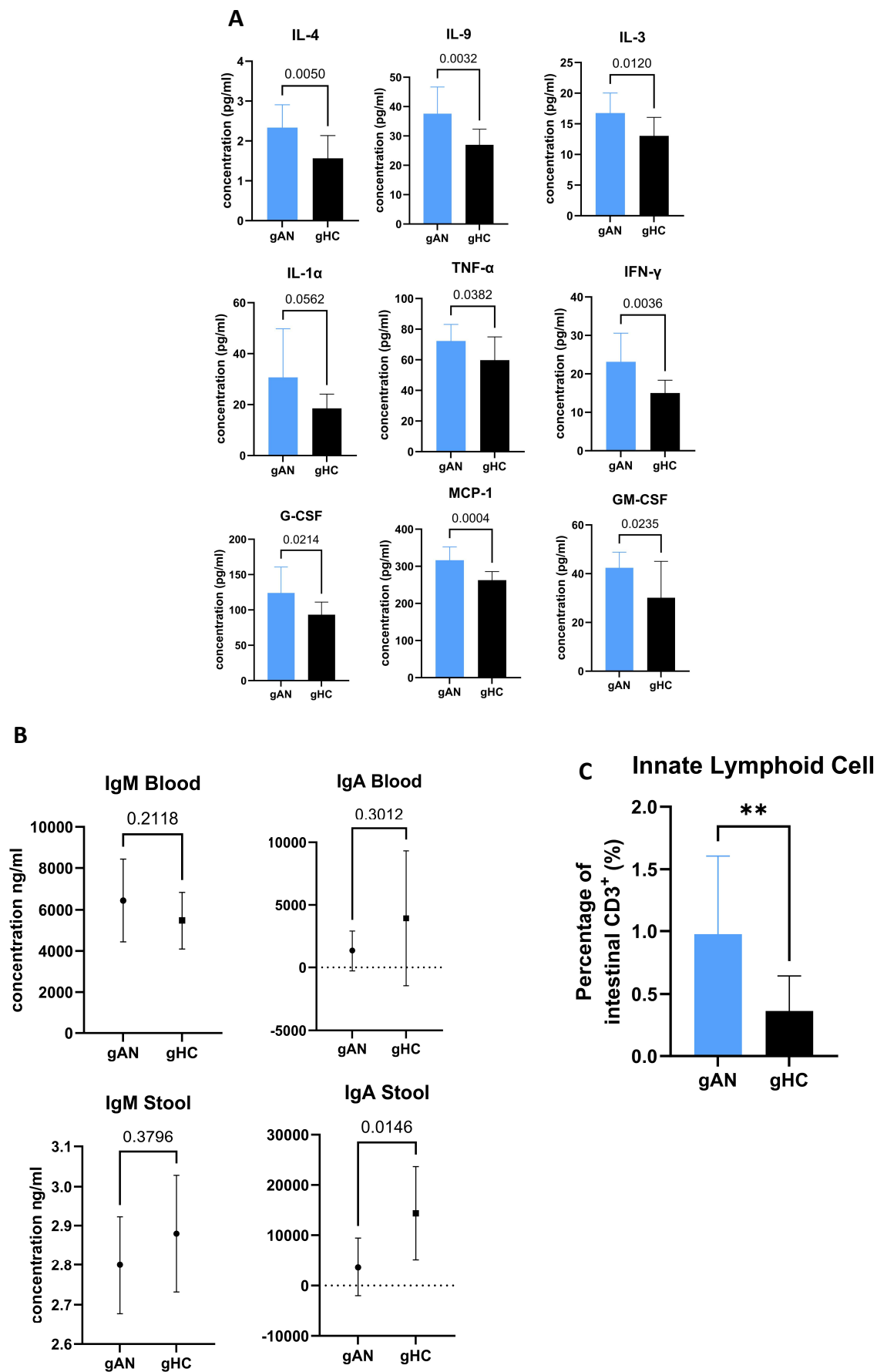

Gabriel-Segard et al. **Fig. 8**

Supplement: Supplementary Material [file KGMI_A_2563701_SM4966.pdf]

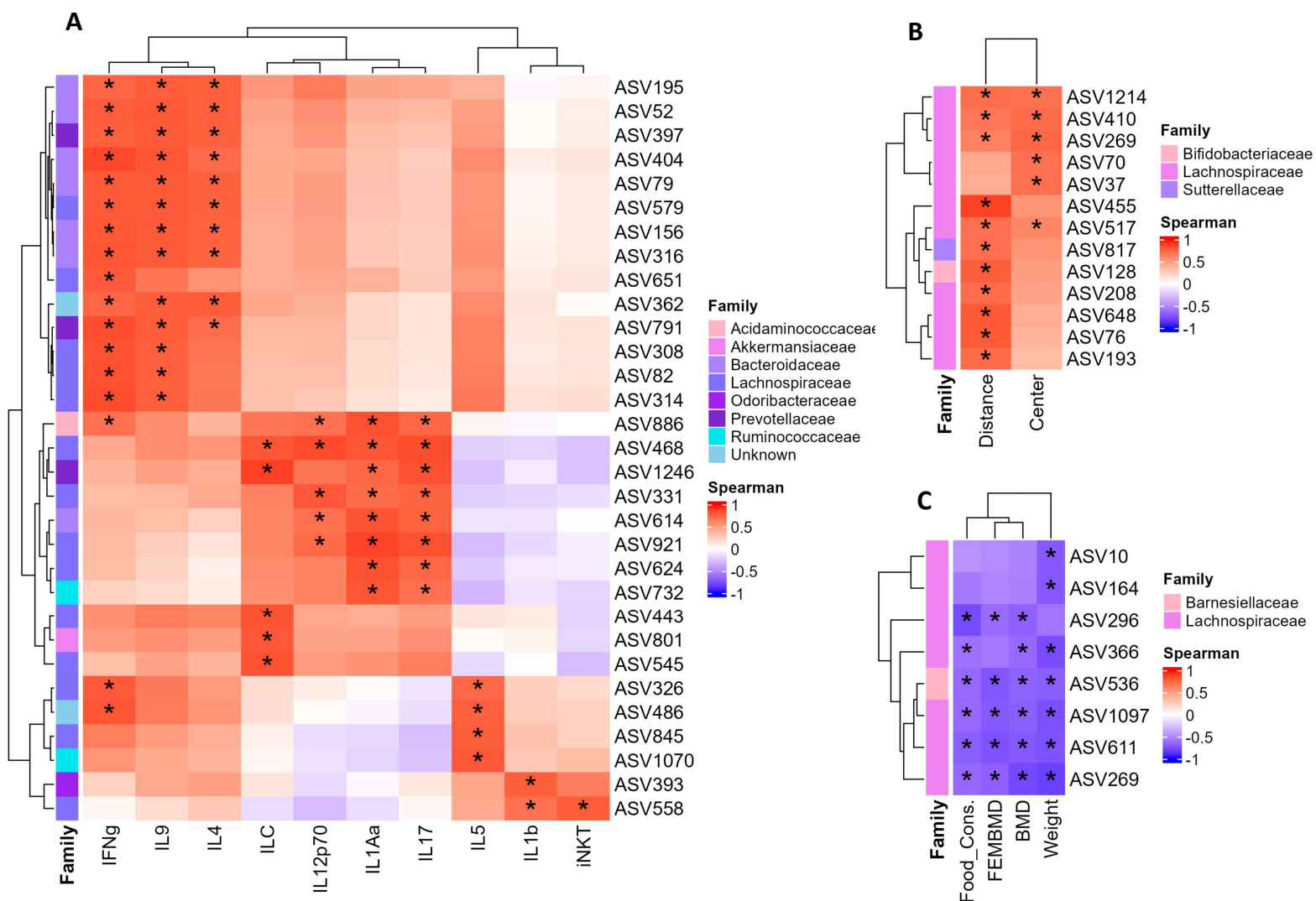

Gabriel-Segard et al. **Fig. 11**

Supplement: Supplementary Material [file KGMI_A_2563701_SM4965.pdf]
