# Supplementary material for: Anorexia nervosa symptoms are induced after specific gut microbiota dysbiosis transfer in germ-free mice
Source: Gut Microbes. 2025 Nov 15;17(1):2563701. doi: 10.1080/19490976.2025.2563701 (PMC12626428; doi:10.1080/19490976.2025.2563701)
Supplement: Supplementary Material [file KGMI_A_2563701_SM4976.pdf]

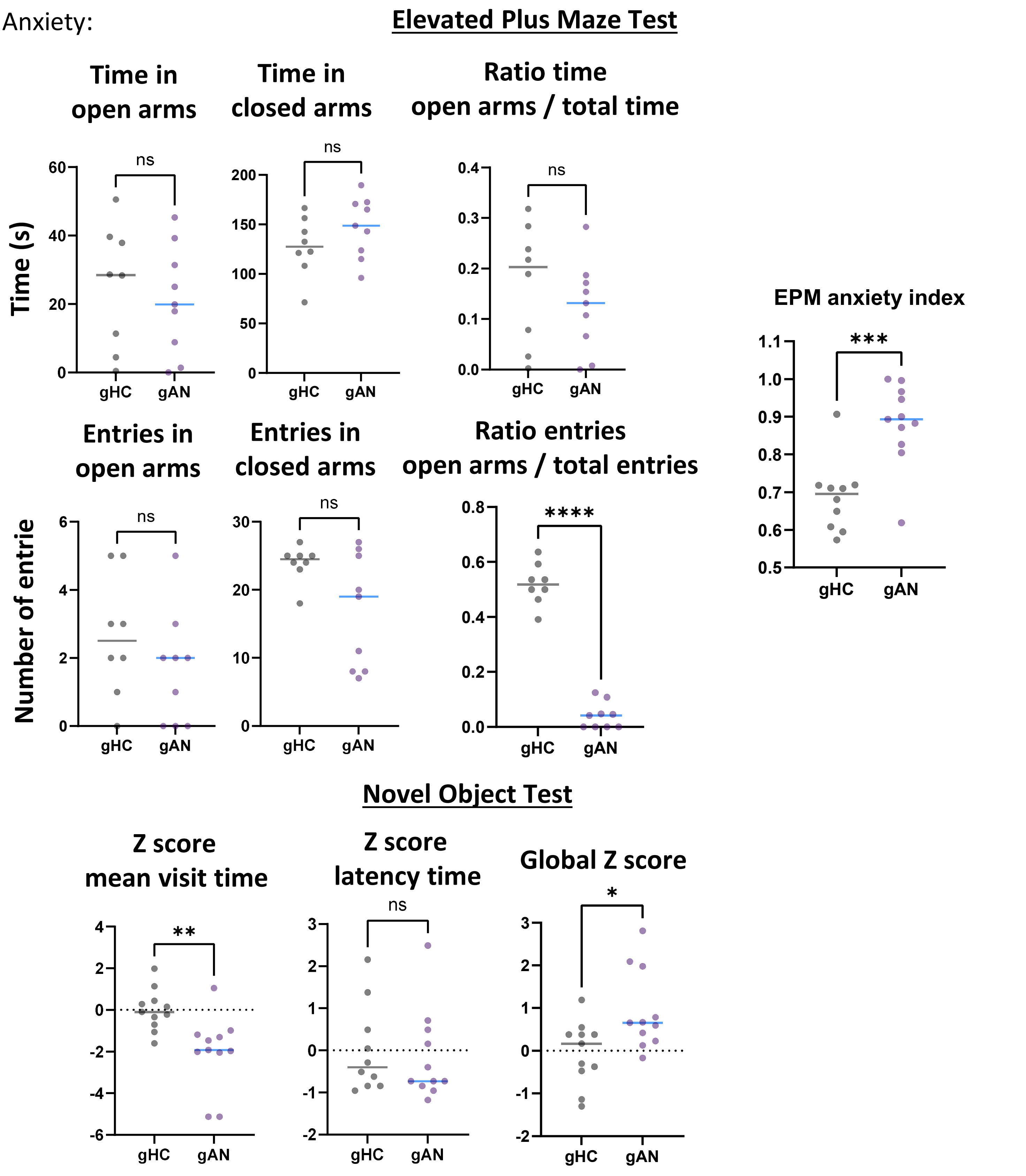

Supp. Fig. 3: detailed dimensions used within elevated plus maze test to calculate the anxiety index as Cohen et al. 2013, and dimensions used from the novel object test to calculate the global Z score.
gAN: gnotobiotic mice from AN group, gHC: gnotobiotic mice from HC group. **: p<0,01; ***: p<0,0005; ****:p<0,0001; ns: non significant.

Gabriel-Segard et al. **Supp Fig. 3**
