# Supplementary material for: Anorexia nervosa symptoms are induced after specific gut microbiota dysbiosis transfer in germ-free mice
Source: Gut Microbes. 2025 Nov 15;17(1):2563701. doi: 10.1080/19490976.2025.2563701 (PMC12626428; doi:10.1080/19490976.2025.2563701)
Supplement: Supplementary Material [file KGMI_A_2563701_SM4974.pdf]

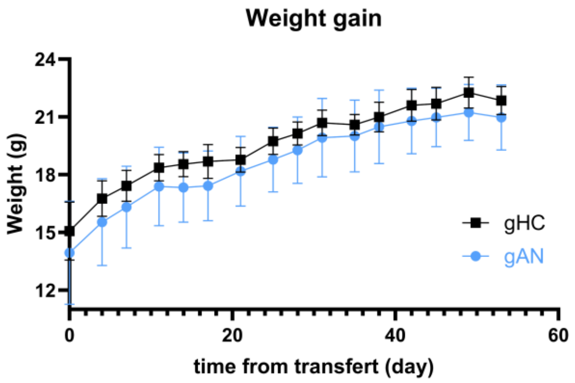

Supplementary Figure 1: Graphic representation of weight of mice by groups along the time of the experiment
