# Supplementary material for: Anorexia nervosa symptoms are induced after specific gut microbiota dysbiosis transfer in germ-free mice
Source: Gut Microbes. 2025 Nov 15;17(1):2563701. doi: 10.1080/19490976.2025.2563701 (PMC12626428; doi:10.1080/19490976.2025.2563701)
Supplement: Supplementary Material [file KGMI_A_2563701_SM4971.pdf]

Physical activity:

Open Field test

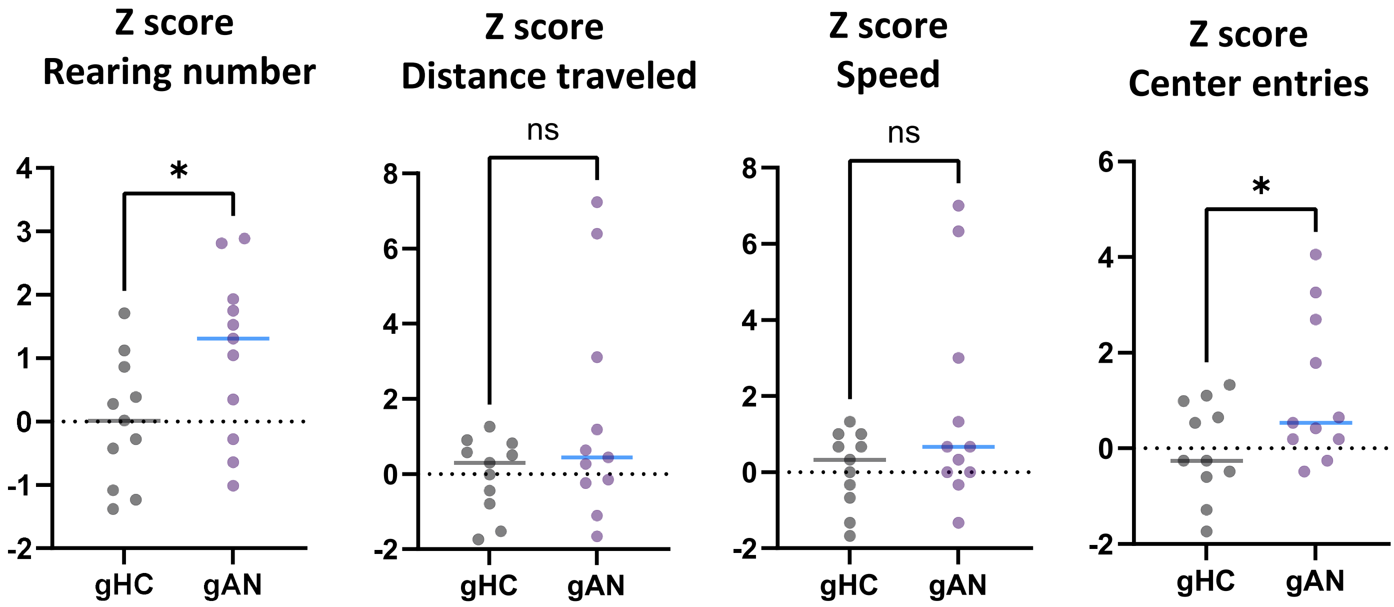

Novel Object test

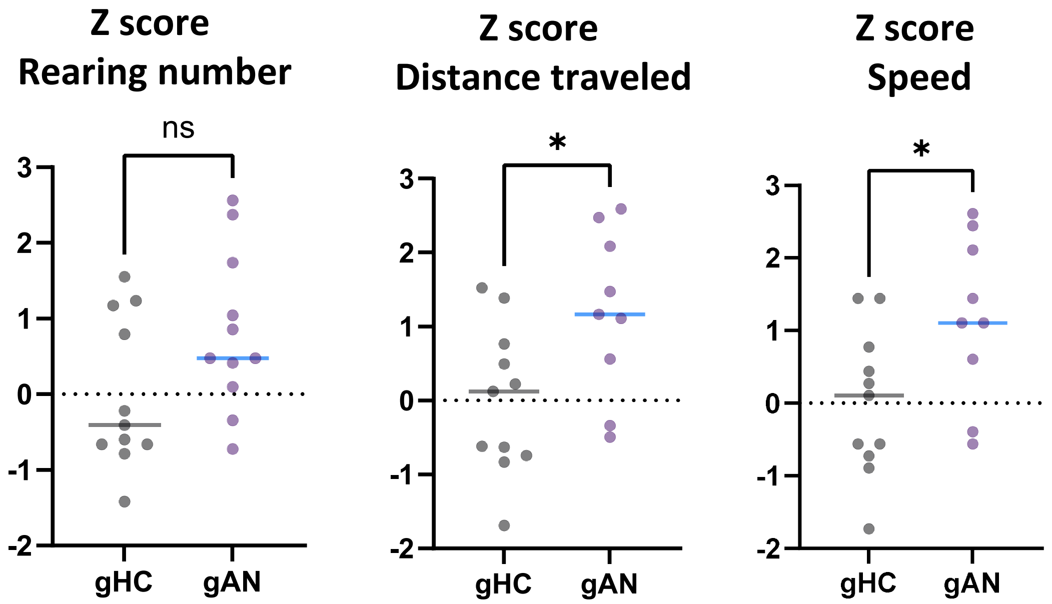

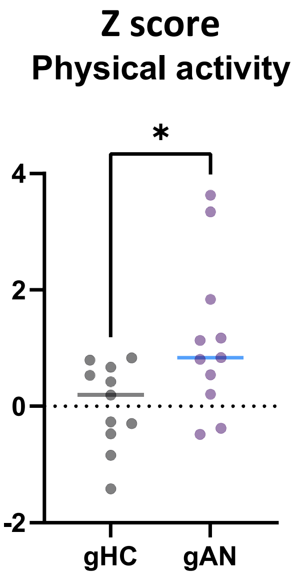

Supp. Fig. 2: detailed dimensions used within open field test and novel object test to calculate the physical activity index.

gAN: gnotobiotic mice from AN group, gHC: gnotobiotic mice from HC group. *: p<0,05

Gabriel-Segard et al. **Supp Fig. 2**
