# Supplementary material for: Anorexia nervosa symptoms are induced after specific gut microbiota dysbiosis transfer in germ-free mice
Source: Gut Microbes. 2025 Nov 15;17(1):2563701. doi: 10.1080/19490976.2025.2563701 (PMC12626428; doi:10.1080/19490976.2025.2563701)
Supplement: Supplementary Material [file KGMI_A_2563701_SM4969.pdf]

Depression

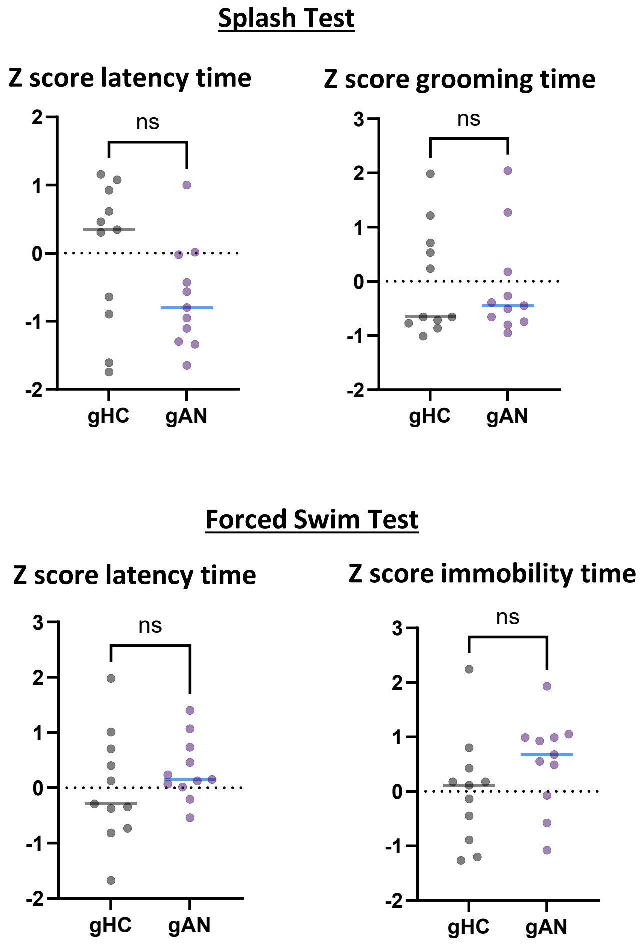

Supp. Fig. 4: detailed dimensions used within the splash test and the forced swim test to evaluate behaviours of pathy and resignation. gAN: gnotobiotic mice from AN group, gHC: gnotobiotic mice from HC group.

Gabriel-Segard et al. **Supp Fig. 4**
