# Supplementary material for: Anorexia nervosa symptoms are induced after specific gut microbiota dysbiosis transfer in germ-free mice
Source: Gut Microbes. 2025 Nov 15;17(1):2563701. doi: 10.1080/19490976.2025.2563701 (PMC12626428; doi:10.1080/19490976.2025.2563701)
Supplement: Supplementary Material [file KGMI_A_2563701_SM4967.pdf]

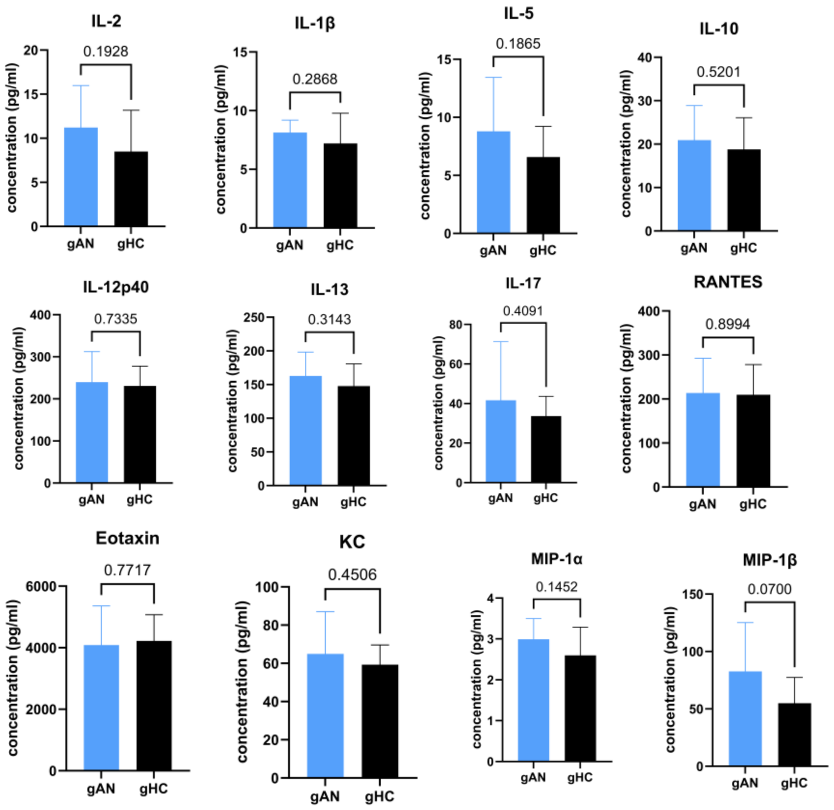

Supplementary figure 3: Graphic representation of analytes without significant difference. Concentration assays were performed in serum by multiplex using a Th17 panel (Bio-Rad Laboratories). gAN: gnotobiotic mice anorexia nervosa group, gHC: gnotobiotic mice healthy controls group

Gabriel-Segard et al. **Supp Fig. 5**
